# Circular RNA circNOL10 Inhibits Lung Cancer Development by Promoting SCLM1‐Mediated Transcriptional Regulation of the Humanin Polypeptide Family

**DOI:** 10.1002/advs.201800654

**Published:** 2018-11-16

**Authors:** Aruo Nan, Lijian Chen, Nan Zhang, Yangyang Jia, Xin Li, Hanyu Zhou, Yihui Ling, Zhishan Wang, Chengfeng Yang, Sijin Liu, Yiguo Jiang

**Affiliations:** ^1^ State Key Laboratory of Respiratory Disease Institute for Chemical Carcinogenesis Guangzhou Medical University Guangzhou 511436 China; ^2^ Department of Toxicology and Cancer Biology Center for Research on Environmental Disease College of Medicine University of Kentucky Lexington KY 40536 USA; ^3^ State Key Laboratory of Environmental Chemistry and Ecotoxicology Research Center for Eco‐Environmental Science Chinese Academy of Sciences Beijing 100085 China

**Keywords:** circular RNA, humanin polypeptide, lung cancer, transcriptional regulation

## Abstract

circNOL10 is a circular RNA expressed at low levels in lung cancer, though its functions in lung cancer remain unknown. Here, the function and molecular mechanism of circNOL10 in lung cancer development are investigated using in vitro and in vivo studies, and it is shown that circNOL10 significantly inhibits the development of lung cancer and that circNOL10 expression is co‐regulated by methylation of its parental gene Pre‐NOL10 and by splicing factor epithelial splicing regulatory protein 1 (ESRP1). circNOL10 promotes the expression of transcription factor sex comb on midleg‐like 1 (SCML1) by inhibiting transcription factor ubiquitination and thus also affects regulation of the humanin (HN) polypeptide family by SCML1. circNOL10 also affects mitochondrial function through regulating the humanin polypeptide family and affecting multiple signaling pathways, ultimately inhibiting cell proliferation and cell cycle progression, and promoting the apoptosis of lung cancer cells, thereby inhibiting lung cancer development. This study investigates the functions and molecular mechanisms of circNOL10 in the development of lung cancer and reveals its involvement in the transcriptional regulation of the HN polypeptide family by SCML1. The results also demonstrate the inhibitory effect of HN on lung cancer cells growth. These findings may identify novel targets for the molecular therapy of lung cancer.

## 1 Introduction

Lung cancer is the main cause of cancer‐related deaths worldwide, and has thus been a focus of cancer research.^1^ Understanding the molecular mechanisms involved in lung cancer development is therefore critical for determining its therapy and prognosis. Increasing evidence suggests the existence of a close association between epigenetics and lung cancer. However, related studies have mainly concentrated on DNA methylation and histone modification,^2^, ^3^, ^4^ and the roles of noncoding RNAs in lung cancer remain uncertain. However, we and others have investigated the association between noncoding RNAs and lung cancer and confirmed an important regulatory function for noncoding RNAs in epigenetics and lung cancer development.^5^, ^6^, ^7^

Increasing numbers of types of noncoding RNAs with different functions have been identified in recent studies, and circular RNAs (circRNAs), which represent a new type of noncoding RNAs, have become a recent topic of research. circRNAs are abundant in the human transcriptome and have diverse biological functions associated with their specific structural features.^8^, ^9^ circRNAs have high stability and show tissue‐specific expression patterns, and are known to be involved in cellular differentiation and pluripotency.^10^, ^11^, ^12^, ^13^ However, the functions of most of the thousands of unique circRNAs discovered to date remain unknown. According to previous reports, most circRNAs are noncoding RNAs, although a few protein‐coding circRNAs have recently been reported.^14^, ^15^ circRNAs have diverse functions and mechanisms of biogenesis, and have been implicated in many different cancers.^16^ Previous studies have also shown close associations between circRNAs and lung cancer, though the specific mechanisms whereby circRNAs may affect carcinogenesis and lung cancer progression are largely unclear.^17^, ^18^

We investigated the functions and molecular mechanisms of circRNAs in lung cancer in experimental studies. Using our high‐throughput circRNA data (Table S1, Supporting Information), we detected the expression of circRNAs in different lung cancer cell lines and in human lung cancer tissue samples, and found that the circRNA circNOL10 (formed by circularization of 6–12 exons of Pre‐NOL10; circBase ID: has_circ_0000977, alias has_circ_001918) was significantly downregulated in lung cancer. Furthermore, Pre‐NOL10 methylation and the splicing factor epithelial splicing regulatory protein 1 (ESRP1) worked together to downregulate circNOL10 expression in lung cancer, associated with significant inhibition of lung cancer development. circNOL10 was mainly located in the nucleus, and bioinformatics analysis revealed that it was involved in transcriptional regulation, rather than protein coding. circNOL10 promoted the expression of sex comb on midleg‐like 1 (SCML1) by inhibiting its ubiquitination. Using chromatin immunoprecipitation sequencing (ChIP‐Seq) and other methods, we showed that SCML1 could regulate the humanin (HN) polypeptide family and that this SCML1‐mediated transcription was promoted by circNOL10. In addition, circNOL10 affected mitochondrial function by promoting the expression of HN. This alteration in mitochondrial function triggered multiple signaling pathways and ultimately inhibited cell proliferation and cell cycle progression and promoted apoptosis of lung cancer cells, thereby significantly inhibiting lung cancer progression.

Most current circRNA studies focus on the role of circRNAs as endogenous competitive RNAs.^19^, ^20^ In contrast, we provide the first evidence for a molecular mechanism whereby circRNAs may affect the transcriptional regulation of genes. We also explored the functions and mechanisms of circRNAs in lung cancer in relation to the HN polypeptide family. The results of this study reveal a new mechanism of lung cancer development and demonstrate the significance of epigenetics in lung cancer.

## 2 Results

### 2.1 circNOL10 Inhibited Lung Cancer Development

**Figure 1:**
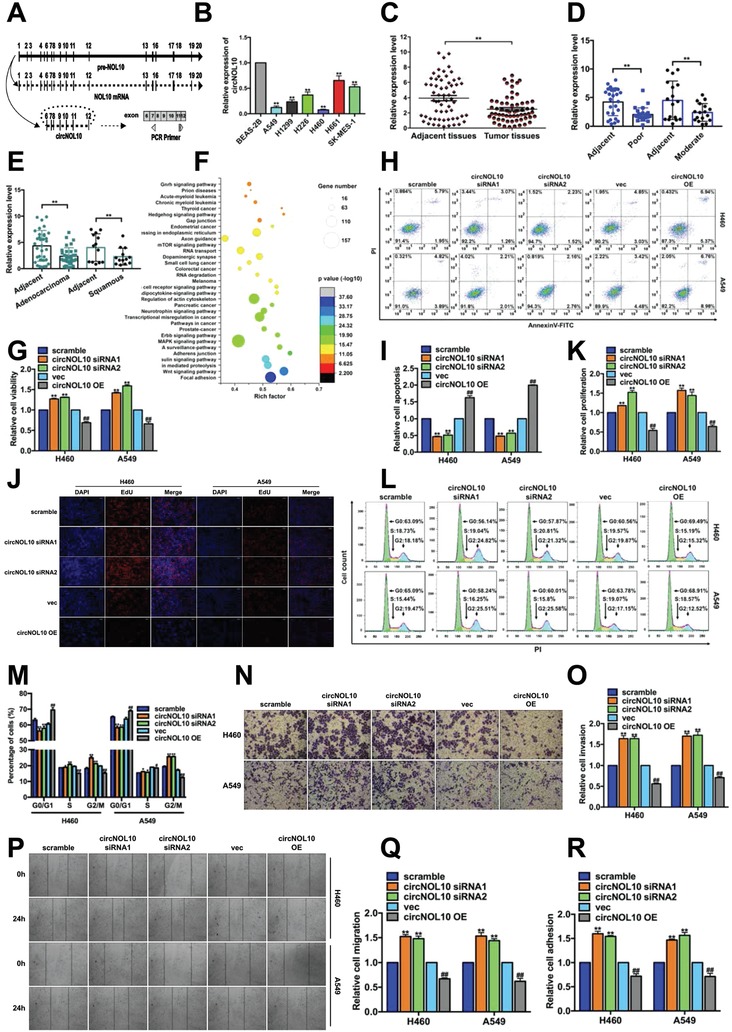
Significant inhibitory effect of circNOL10 on lung cancer development in vitro. A) Pre‐NOL10 is the parental gene of circNOL10, and NOL10 mRNA is the mature linear RNA. We designed PCR primers to span the back‐splice junction to ensure the specificity of circNOL10 detection. B) circNOL10 in normal bronchial epithelial cells (BEAS‐2B) and lung cancer cell lines (A549, H1299, H226, H460, H661, and SK‐MES‐1) by qPCR. Mean ± SD, *n* = 3; unpaired *t*‐test, **P < 0.01. C) circNOL10 in tumor tissues and adjacent tissues by qPCR. Mean ± SD, *n* = 61; ANOVA, **P < 0.01. D) circNOL10 detection in poorly and moderately differentiated lung cancers using qPCR. Mean ± SD, *n* = 3; ANOVA, **P < 0.01. E) circNOL10 detection in adenocarcinoma and squamous cell carcinoma using qPCR. Mean ± SD, *n* = 3; ANOVA, **P < 0.01. F) circNOL10‐sponged miRNA pathway analysis by OriginPro2016. The *Y*‐axis indicates pathways; the *X*‐axis indicates enrichment factor (ratio of differentially expressed genes enriched in the pathways to the number of annotated genes in the corresponding pathways); size of the point indicates the number of differentially expressed genes in the corresponding pathways (gene number); color of the point refers to the *P* value range. G) circNOL10 function in tumor cell viability detected with CCK8 assay. H,I) circNOL10 function in tumor cell apoptosis detected by flow cytometry (FCM). Cells stained with AnnexinV‐fluorescein isothiocyanate (FITC) and propidium iodide (PI) for apoptosis detection. J,K) circNOL10 function in tumor cell proliferation detected by EdU assay. Nuclei were stained with DAPI. Combined reaction with EdU and DAPI indicated cells in S phase. L,M) circNOL10 function in tumor cell cycle detected with FCM. Cells stained with PI. Cell count indicates the number of cells in different cell cycle phases. N,O) circNOL10 function in tumor cell invasion detected with transwell assay. P,Q) circNOL10 function in tumor cell metastasis detected with scratch assay. R) circNOL10 function in tumor cell adhesion detected with adhesion assay. G,I,K,M,O,Q,R) Mean ± SD, *n* = 3; unpaired *t*‐tests; compared with the scramble group, *P < 0.05, **P < 0.01; compared with the vec group, ^#^ *P* < 0.05, ^##^ *P* < 0.01.

circNOL10 was shown to be a circRNA formed by cyclization of exons 6–12 of Pre‐NOL10. A linear RNA (NOL10 mRNA) was also generated during the formation of circNOL10 (UCSC Genome Browser). Divergent primers spanning the backsplicing site were designed and used to detect circNOL10 expression (**Figure** **1**A and Figure SA,B, Supporting Information) in six lung cancer cell lines (A549, H1299, H226, H460, H661, and SK‐MES‐1) and in noncancerous bronchial epithelial BEAS‐2B cells (control). circNOL10 expression levels were significantly decreased in all six lung cancer cell lines, and were lowest in H460 and A549 cells (decreased by 13‐fold and 8‐fold, respectively) (Figure 1B). circNOL10 expression was also detected in 61 pairs of cancerous and paracancerous lung tissue samples, and levels were shown to be significantly lower in the cancer tissues in 41 of the 61 paired samples (Figure S1C, Supporting Information). The decrease in circNOL10 expression in lung cancer compared with control tissues was statistically significant (Figure 1C). circNOL10 expression levels were also significantly lower in poorly and intermediately differentiated lung cancers, and the decreases in lung squamous cell carcinoma and lung adenocarcinoma tissues were both significant (Figure 1D,E).

We further examined the function of circNOL10 in cell lines in vitro by silencing its expression in H460 and A549 cells using three types of small interfering (si)RNAs. The silencing efficiencies of siRNA1 and siRNA2 were both more than twice that of the scrambled sequence, and also significantly higher than that of siRNA3 (Figure S1D, Supporting Information). A vector overexpressing circNOL10 was designed (Figure S1E, Supporting Information) and resulted in more than tenfold increases in expression levels in both H460 and A549 cells, compared with blank vector (vec) (Figure S1F, Supporting Information). We investigated the specificities of the silencing and overexpression (OE) experiments for circNOL10 and demonstrated that the siRNAs and overexpression vector silenced and overexpressed circNOL10, respectively, in H460 and A549 cells, while Pre‐NOL10 and NOL10 mRNA levels were unaffected (Figure S1G, Supporting Information).

H460 and A549 cells were transiently transfected with circNOL10 siRNA1 and siRNA2 to silence circNOL10 expression, and with circNOL10 OE vector to overexpress circNOL10 for in vitro functional studies. Moreover, we analyzed circNOL10‐sponged miRNA in relation to regulatory RNA motifs and elements (RegRNA; http://regrna.mbc.nctu.edu.tw/index1.php) (Table S2, Supporting Information) and pathways (mirPath v.3; http://snf‐515788.vm.okeanos.grnet.gr) (Table S3, Supporting Information), and revealed that circNOL10 was involved in multiple pathways associated with tumor development (Figure 1F). We further examined the effects of circNOL10 on cellular behaviors or processes, including cell viability (Figure 1G), apoptosis (Figure 1H,I), proliferation (Figure 1J,K), cell cycle (Figure 1L,M and Figure S1H,I, Supporting Information), invasion (Figure 1N,O), migration (Figure 1P,Q), and adhesion (Figure 1R). Compared with transfection with a scrambled sequence, transfection of H460 cells and A549 cells with circNOL10 siRNA1 or siRNA2 significantly increased cell viability, proliferation, invasion ability, migration, and adhesion, as well as the proportion of cells in S and G2/M phases, and significantly decreased the proportion of cells undergoing apoptosis and in G0/G1 phase. Cells transfected with circNOL10 OE showed opposite changes to those seen in the gene‐silencing groups, compared with blank vector (vec).

**Figure 2:**
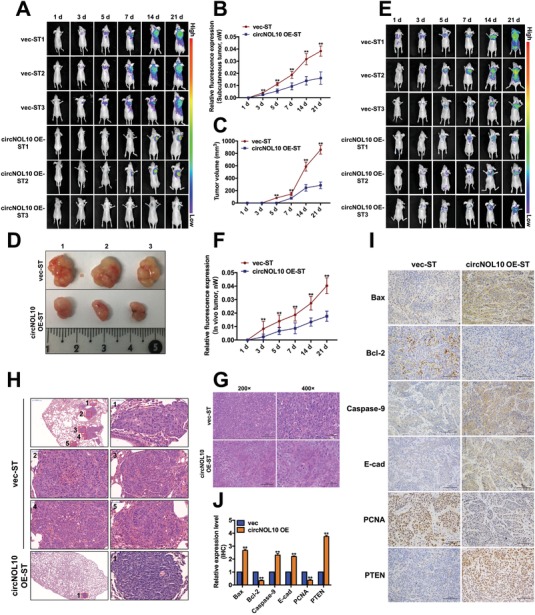
Significant inhibitory effect of circNOL10 on lung cancer development in vivo. A) Nude mice injected subcutaneously with circNOL10 stable‐overexpression (circNOL10 OE‐ST) or control cells (vec‐ST). Subcutaneous tumor fluorescence intensities were detected in vivo using an in vivo small animal imaging system at 1, 3, 5, 7, 14, and 21 d, indicating tumor growth. B) Relative fluorescence intensities in subcutaneous tumor‐bearing nude mice at 1, 3, 5, 7, 14, and 21 d. C) Tumor sizes in subcutaneous tumor‐bearing nude mice at 1, 3, 5, 7, 14, and 21 d. D) Subcutaneous tumors removed from nude mice at 21 d. E) Nude mouse models were created by caudal vein injection of cells (circNOL10 OE‐ST and vec‐ST) and fluorescence intensities were detected in vivo at 1, 3, 5, 7, 14, and 21 d using a small animal imaging system. F) Relative fluorescence intensities in caudal vein injection nude mice at 1, 3, 5, 7, 14, and 21 d. G) HE staining of subcutaneous tumors at ×200 and ×400. H) HE staining of lung tissues at ×40 and ×400. I) Immunohistochemical assays of six tumor markers (Bax, Bcl‐2, caspase‐9, E‐cadherin, PCNA, and PTEN) in subcutaneous tumors (×200). J) Quantitative immunohistochemical analysis of six tumor markers. B,C,F) *n* = 3, mean ± SD; ANOVA, **P < 0.01. J) *n* = 3, mean ± SD; unpaired *t*‐test, **P < 0.01.

We also explored the function of circNOL10 in vivo by subcutaneous injection into tumor‐bearing nude mice. A green fluorescent protein (GFP) tagged circNOL10 OE vector (Figure S1J, Supporting Information) and GFP‐tagged blank vector were constructed and packaged in lentiviruses, and then stably transfected into the cell lines. Their overexpression efficiency was detected with quantitative polymerase chain reaction (qPCR). Compared with H460 cells stably transfected with blank vector (vec‐ST) as a control, circNOL10 expression was increased 12‐fold in cells stably transfected with overexpression vector (circNOL10 OE‐ST) (Figure S1K, Supporting Information). We investigated the function of circNOL10 in nude mice with subcutaneous tumors using in vivo imaging (**Figure** **2**A) to detect the fluorescence intensities (Figure 2B) and sizes of the subcutaneous tumors (Figure 2C). The tumors were then removed and observed at 21 d after injection of the transfected cells (Figure 2D). The fluorescence intensities at different time points were significantly lower in the circNOL10 OE‐ST group compared with the control vec‐ST group, while tumors also appeared later in the circNOL10 OE‐ST compared with the vec‐ST group. The tumors were solid, and tumors in the circNOL10 OE‐ST group were significantly smaller than those in the vec‐ST group. We created a mouse lung cancer xenograft model by injection of vec‐ST and circNOL10 OE‐ST cells via the caudal vein (Figure 2E). The fluorescence intensities and tumor growth rates at different time points were significantly lower in the circNOL10 OE‐ST compared with the vec‐ST group, as shown with in vivo imaging (Figure 2F).

Hematoxylin and eosin (HE) staining of the subcutaneous tumors showed that the nuclear: cytoplasmic ratio and nuclear atypia were both reduced in the circNOL10 OE‐ST compared with the control group, suggesting that circNOL10 overexpression reduced the degree of malignancy (Figure 2G). HE staining also revealed fewer lung‐tumor foci in the circNOL10 OE‐ST compared with the vec‐ST group, and a lower degree of malignancy (Figure 2H). We detected the expression levels of six tumor biomarkers by immunohistochemical assay. Expression levels of the proapoptotic protein Bax and caspase‐9 were significantly increased in the circNOL10 OE‐ST group compared with the vec‐ST group, while levels of the apoptosis‐inhibitory protein Bcl‐2 were significantly decreased. Furthermore, expression of the invasion‐inhibitory marker E‐cadherin and the tumor suppressor phosphatase and tensin homolog deleted on chromosome 10 (PTEN) were significantly increased, while the proliferation marker proliferating cell nuclear antigen (PCNA) was significantly decreased by circNOL10 overexpression (Figure 2I,J). This in vivo study thus confirmed the inhibitory effect of circNOL10 on tumor development.

### 2.2 Pre‐NOL10 Methylation and Splicing Factor ESRP1 Cooperatively Inhibited circNOL10 Expression in Lung Cancer Cells

**Figure 3:**
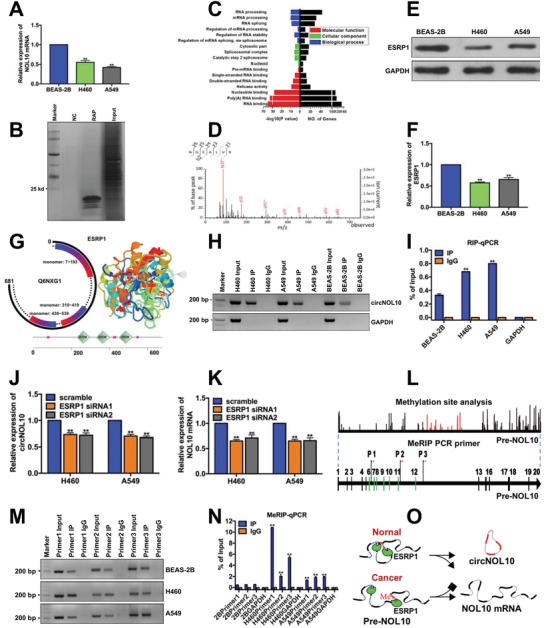
Causal exploration of significant circNOL10 downregulation in lung cancer cells. A) N0L10 mRNA expression in BEAS‐2B, H460, and A549 cells determined by qPCR. B) Silver staining of purified interaction proteins in Pre‐NOL10 RAP experiment. NC, scramble control group; RAP, protein group obtained by RNA antisense purification; and Input, total protein group. C) GO analysis of Pre‐NOL10 RAP‐MS results. Molecular function, cellular component, and biological process represent three aspects of GO analysis. Larger −log10 (*P* value) indicates more significant enrichment and more differentially expressed genes (No. of genes). GO analysis indicated that the interaction proteins were involved in multiple RNA splicing pathways. D) Mass spectrogram of ESRP1 protein. E,F) Semi‐quantitative analysis of ESRP1 protein expression in BEAS‐2B, H460, and A549 cells detected with western blot. G) ESRP1 protein domain analysis (http://smart.embl‐heidelberg.de) (Q6NXG1 is an alias for ESRP1). Three monomer positions of ESRP1 are shown. RRM indicates the three functional domains in the ESRP1 protein secondary structure. H,I) ESRP1 RIP‐PCR and RIP‐qPCR results for circNOL10 and GAPDH in BEAS‐2B, H460, and A549 cells. J) circNOL10 expression in H460 and A549 cells after silencing ESRP1, determined with qPCR. K) NOL10 mRNA expression in H460 and A549 cells after silencing ESRP1, determined by qPCR. L) Pre‐NOL10 methylation site prediction and primer design for m6A MeRIP PCR. M,N) MeRIP‐PCR and MeRIP‐qPCR results of m6A in BEAS‐2B, H460, and A549 cells. O) Causal diagram of downregulation of circNOL10 in lung cancer cells. A,F,I,J,K,N) *n* = 3, mean ± SD, unpaired *t*‐test, **P < 0.01.

Given that downregulation of circNOL10 in lung cancer cells had a significant synergistic effect on lung cancer development, we investigated the reasons for this downregulation to improve our understanding of the functions and associated molecular mechanisms of circNOL10. Linear mature NOL10 mRNA and circNOL10 are both derived from the Pre‐NOL10 gene. NOL10 mRNA expression levels were reduced 1.8‐fold and 2.4‐fold in H460 and A549 cells, respectively, compared with noncancerous BEAS‐2B cells (**Figure** **3**A), whereas circNOL10 levels were decreased 13‐fold and 8‐fold, respectively (Figure 1B). circNOL10 expression was thus reduced more significantly than NOL10 mRNA in lung cancer cells.

To clarify the regulatory mechanism of circNOL10 formation, we analyzed the genomic elements likely to affect circNOL10 circularization, and then performed genomic sequencing of the flanking intron regions. There were a few mutation sites, but none in the circularization‐associated elements (Figure S2A, Supporting Information). This suggested that low circNOL10 expression in tumor cells was unlikely to be due to mutation of the NOL10 gene.

We designed RNA antisense purification (RAP) probes targeting Pre‐NOL10 and performed RAP assays to detect proteins binding to Pre‐NOL10. Silver staining showed numerous proteins interacting with Pre‐NOL10. The molecular weights of most of the purified proteins were <25 kD (Figure 3B). The purified proteins were identified using mass spectrometry (MS) and subjected to gene ontology (GO) analysis (http://www.geneontology.org). Pre‐NOL10‐binding proteins were shown to have important functions in RNA splicing (Figure 3C). Based on the MS results and on previous studies suggesting that RNA splicing factors can regulate the formation of specific circRNAs,^13^ we identified ESRP1 as a typical splicing factor involved in the formation of circRNAs. In the current study, we therefore investigated the role of ESRP1 in circNOL10 formation. MS confirmed the expression of ESRP1 protein (Figure 3D), and western blotting showed that ESRP1 was significantly downregulated in A549 and H460 cells compared with BEAS‐2B cells (Figure 3E,F). Structural analysis of ESRP1 (http://smart.embl‐heidelberg.de) showed that it comprised three monomers, the positions of which corresponded to the positions of the functional domain displayed in the secondary structure (Figure 3G). circNOL10 expression levels in the ESRP1 RNA immunoprecipitation (RIP) products of H460 and A549 cells were higher than in BEAS‐2B cells, as demonstrated with RIP‐PCR, indicating that ESRP1 had a higher binding capacity for circNOL10 in H460 and A549 cells than in BEAS‐2B cells (Figure 3H,I). We silenced ESRP1 in H460 and A549 cells (Figure SB, Supporting Information) using siRNA1 and siRNA2 to clarify its effect on the formation of circNOL10 and NOL10mRNA. circNOL10 and NOL10 mRNA levels were both significantly downregulated by silencing ESRP1 (Figure 3J,K). In addition to splicing factors, RNA splicing is also regulated by m6A methylation.^21^ We therefore analyzed the methylation sites in Pre‐NOL10 (https://www.ncbi.nlm.nih.gov/). We identified numerous RNA methylation sites in Pre‐NOL10 and selected three in the exons near the cyclization region for further study. We designed specific primers (Figure 3L) for N6‐methyladenosine RIP PCR (m6A MeRIP‐PCR) and showed that the methylation levels of all three sites were significantly increased in H460 and A549 cells compared with BEAS‐2B cells (Figure 3M,N). RNA methylation is known to be a switch regulating RNA structure, and hypermethylation opens nucleic acid bonds and thus affects the formation of circRNAs rather than linear mRNAs.^22^ We therefore supposed that hypermethylation of Pre‐NOL10 in H460 and A549 cells inhibited the formation of circNOL10. Overall, these results suggested that the low circNOL10 expression levels relative to NOL10 mRNA in lung cancer cells may be a consequence of co‐regulation by ESRP1 and Pre‐NOL10 methylation (Figure 3O).

### 2.3 circNOL10 Directly Promoted Expression of the Transcription Factor SCML1 in Lung Cancer Cells

**Figure 4:**
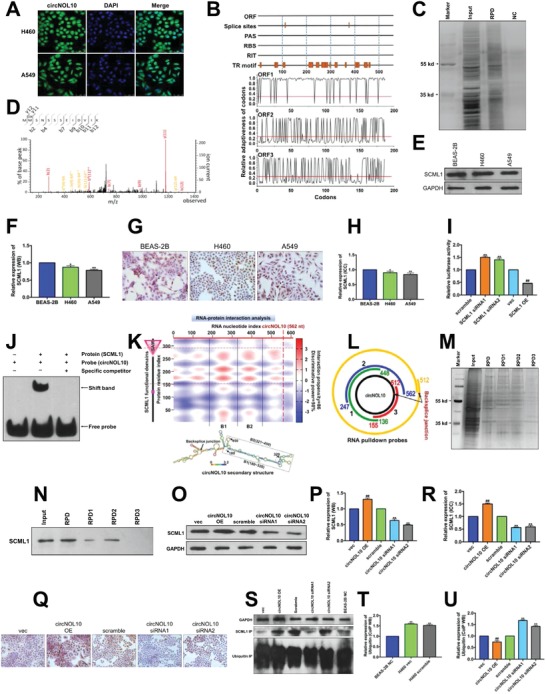
Mechanism of circNOL10 in regulating transcription factor SCML1. A) Distribution of circNOL10 in H460 and A549 cells determined by FISH. circNOL10 group, using specific probe; DAPI staining indicates nuclei; merge represents an overlay figure. B) circNOL10 nucleic acid sequence indicating open reading frames (ORF), splice sites, polyadenylation sites (PAS), ribosome binding site (RBS), Rho‐independent terminator (RIT), transcriptional regulatory motif (TR motif), and codons. There were two splice sites and several transcriptional regulatory motifs. The structure suggested that circNOL10 was unlikely to encode a protein. C) Silver staining of proteins obtained by RNA pull‐down (RPD). A specific probe against circNOL10 was designed for RPD. Input group, total protein group; RPD group, pull‐down group; NC, scramble control group. D) Mass spectrogram of SCML1 protein. E,F) Semi‐quantitative analysis of SCML1 protein expression in BEAS‐2B, H460, and A549 cells detected with western blot. G,H) Quantitative analysis of SCML1 protein expression in BEAS‐2B, H460, and A549 cells detected with immunocytochemistry assay. I) Construction of a dual luciferase reporter vector targeting circNOL10 and relative fluorescence intensity after SCML1 silencing or overexpression. J) Interaction between circNOL10 and SCML1 detected by RNA‐EMSA. Protein (SCML1), prokaryotically expressed SCML1 protein; probe (circNOL10), circNOL10‐specific RNA EMSA probe; specific competitor, nonmutable competitive probe. K) Bioinformatics analysis of interaction region and intensity between circNOL10 and SCML1, and identification of domains and matched binding sites in relation to circNOL10 secondary structure analysis. SAM, SCML1 domain; B1, Binding1; B2, Binding2. L) Three truncated RPD probes and a specific RNA pull‐down probe were designed to capture circNOL10‐interacting proteins based on the matching sites. M) Silver staining of proteins obtained by RNA pull‐down assay using circNOL10‐ specific RNA pull‐down probe and three truncated probes. N) Qualitative analysis of SCML1 protein expression in RNA pull‐down products with different probes, detected with western blot. O,P) Semi‐quantitative analysis of SCML1 protein expression in H460 cells after circNOL10 silencing or overexpression, detected with western blot. Q,R) Quantitative analysis of SCML1 protein expression in H460 cells after circNOL10 silencing or overexpression, detected with ICC. S–U) Semi‐quantitative analysis of SCML1 and ubiquitin protein co‐immunoprecipitation products in H460 and BEAS‐2B cells after circNOL10 silencing or overexpression, detected with western blot. F,H,I,P,R,T,U) n = 3, mean ± SD; unpaired t‐test; compared with BEAS‐2B, BEAS‐2B NC or scramble group, *P < 0.05, **P < 0.01; compared with vec group, ^##^P < 0.01.

We further explored the molecular mechanism responsible for the inhibitory effect of circNOL10 in lung cancer development by determining its subcellular localization in H460 and A549 cells with fluorescence in situ hybridization (FISH). circNOL10 was expressed in both the cytoplasm and nucleus, but levels were significantly higher in the nucleus (**Figure** **4**A). Bioinformatics analysis of circNOL10 showed that it lacked an open reading frame, polyadenylation sites, ribosome‐binding site, and an Rho‐independent terminator, but included human splicing sites and a transcriptional regulatory motif (http://www.jcat.de/Start.jsp;http://regrna2.mbc.nctu.edu.tw/detection_output.php). Some rare codons were found, and the relative adaptiveness of most codons was higher than the mean codon usage (red line, Figure 4B). Based on these data and its predominant expression in the nucleus, we considered that circNOL10 was unlikely to encode a protein and may be involved in transcriptional regulation.

We investigated transcriptional factors potentially regulated by circNOL10 by RNA pull‐down assay of circNOL10‐binding proteins followed by silver staining (Figure 4C). MS analysis identified one particularly abundant transcription factor (Figure S3A, Supporting Information) as SCML1 (Figure 4D). SCML1 was downregulated in lung cancer cells (H460 and A549) compared with control BEAS‐2B cells, as shown by western blot (Figure 4E,F) and confirmed by immunocytochemistry (Figure 4G,H).

We studied the interaction between circNOL10 and SCML1 by luciferase reporter gene assay. We constructed SCML1‐silencing and overexpression vectors (Figure S3B,C, Supporting Information), and circNOL10 reporter gene vectors and co‐transfected them into lung cancer cells. Compared with cells transfected with scrambled sequences, relative fluorescence intensity was significantly increased in SCML1‐silenced cells, and was significantly decreased by SCML1 overexpression (Figure 4I). These results suggested an association between circNOL10 and SCML1. To verify the direct interaction between circNOL10 and SCML1, we constructed a prokaryotic SCML1‐overexpression vector and obtained purified SCML1 protein (Figure S3D, Supporting Information). We then tested the direct interaction between circNOL10 and SCML1 by RNA electrophoretic mobility shift assay (EMSA). A shifted band was detected in the SCML1 and circNOL10 EMSA probe group, but not in the circNOL10 or nonmutant probe groups (Figure 4J). This confirmed a direct interaction between circNOL10 and SCML1.

The interacting regions of circNOL10 and SCML1 were predicted using catRAPID tool (http://service.tartaglialab.com/grant_submission/catrapid_graphic). The interaction propensity was 86% and discriminative power was 98%, with two obvious binding regions, Binding1 (B1) and Binding2 (B2). Secondary structure analysis of circNOL10 (http://rna.urmc.rochester.edu/RNAstructureWeb/) showed RNA functional domains in the B1 and B2 binding regions. Analysis of SCML1 functional domains (http://smart.embl‐heidelberg.de) showed amino acids 255–324 of SCML1 included a sterile alpha motif (SAM) domain, located in the binding regions predicted by catRAPID (Figure 4K). The full‐length circNOL10 probe was truncated to three segments (Figure 4L), and the full‐length and truncated probes were then used in RNA pull‐down assays to identify the specific locations of the binding regions. RPD, RPD1, and RPD2 pulled down abundant proteins, but RPD3 failed to pull down any proteins (Figure 4M). The proteins obtained by RNA pull‐down were further detected and analyzed with western blot, which confirmed the positive results for RPD, RPD1, and RPD2, but negative results for RPD3 (Figure 4N). These findings confirmed that circNOL10 interacted with the SCML1 B1 and B2 regions.

We detected SCML1 expression in H460 cells after silencing or overexpression of circNOL10 by western blot, to investigate the regulatory interaction between circNOL10 and SCML1. SCML1 expression was increased by overexpression of circNOL10, and decreased by circNOL10 silencing (Figure 4O,P). circNOL10 overexpression was also associated with increased SCML1 expression and silencing of circNOL10 with decreased SCML1 expression according to immunocytochemistry (Figure 4Q,R). These results indicated circNOL10 promoted SCML1 expression in lung cancer cells.

High levels of protein ubiquitination have been reported to accelerate their degradation, while RNAs can promote the expression of RNA‐binding proteins by inhibiting protein ubiquitination.^23^, ^24^ To confirm if circNOL10 increased SCML1 expression by inhibiting ubiquitination, we identified and analyzed the ubiquitination sites in SCML1 (http://bdmpub.biocuckoo.org/prediction.php) (Figure S3E, Supporting Information). We then silenced or overexpressed circNOL10 in H460 cells and performed SCML1 co‐immunoprecipitation assays in BEAS‐2B cells (Figure S3F, Supporting Information). We detected SCML1 and ubiquitin with western blot and showed that the degree of SCML1 ubiquitination was significantly higher in H460 compared with BEAS‐2B cells. Together with the fact that SCML1 was downregulated in cancer cells, we hypothesized that ubiquitination was involved in regulating the expression of SCML1 in lung cancer cells. Ubiquitin expression was significantly decreased by circNOL10 overexpression and increased by silencing circNOL10 (Figure 4S–U). All these results suggested that circNOL10 inhibited SCML1 ubiquitination to increase its expression in lung cancer cells.

### 2.4 circNOL10 Promoted the Transcriptional Regulatory Effect of SCML1 on HN

**Figure 5:**
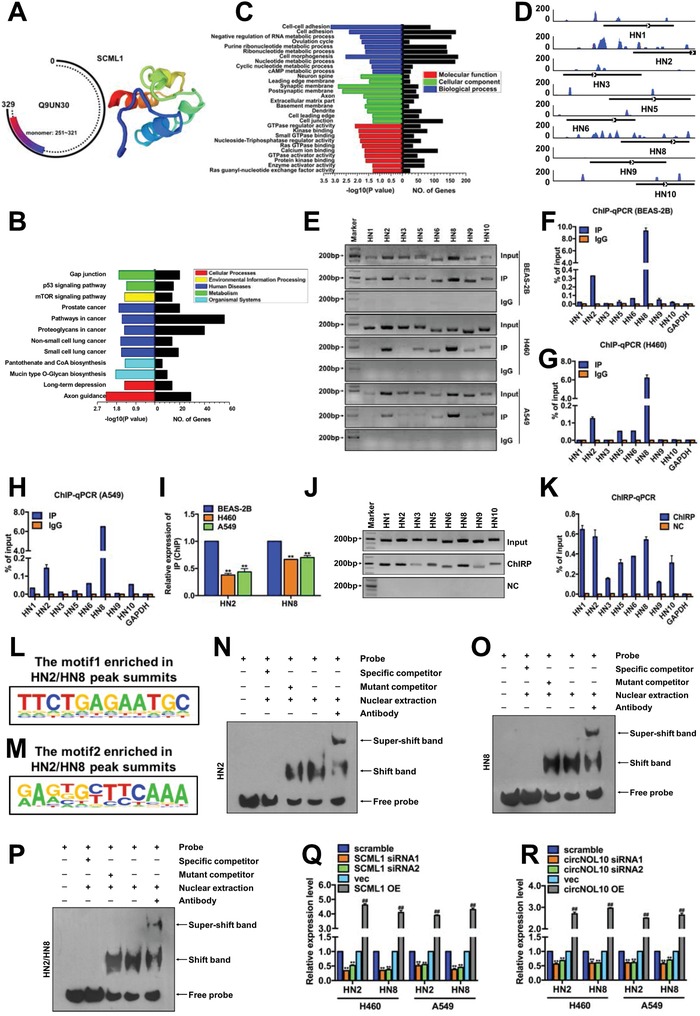
Mechanism of transcriptional regulation of HN polypeptide family by SCML1. A) SCML1 protein domain analysis (http://smart.embl‐heidelberg.de) (Q9UN3 is an alias for SCML1). Monomer: 251–321 was a monomer of SCML1. B) KEGG analysis of SCML1 ChIP‐Seq results. Cellular process, environmental information processing, human diseases, metabolism, and organismal systems are five aspects of KEGG analysis. Larger −log (*P* value) indicates more significant enrichment and more differentially expressed genes (No. of genes). C) GO analysis of SCML1 ChIP‐Seq results. Molecular function, cellular component, and biological process represent three aspects of GO analysis. Larger value of −log10 (*P* value) indicates more significant enrichment and more differentially expressed genes (No. of genes). D) Peaks of HN polypeptide family in ChIP‐Seq plot. E–I) Detection of HN polypeptide family in SCML1 ChIP products (BEAS‐2B, H460, and A549 cells) with PCR and qPCR. J,K) Detection of HN polypeptide family in circNOL10 ChIRP products (BEAS‐2B, H460, and A549 cells) with PCR and qPCR. L,M) SCML1 motif sequences enriched in HN2/HN8 peaks. N–P) DNA EMSA. Probe, biotinylated circNOL10‐specific EMSA probe; specific competitor, wild‐type competitive probe; mutant competitor, mutant competitive probe; nuclear extraction, nuclear protein extract from H460 cells; antibody, SCML1‐ specific antibody. Q) HN2 and HN8 expression in H460 and A549 cells detected with qPCR after SCML1 silencing or overexpression. R) HN2 and HN8 expression in H460 and A549 cells detected with qPCR after circNOL10 silencing or overexpression. I,Q,R) *n* = 3, mean ± SD; unpaired *t*‐test; **P < 0.01 compared with BEAS‐2B or scramble group; ^##^ *P* < 0.01 compared with vec group.

We analyzed the tertiary structure of SCML1 and identified amino acids (aa) 251–321 as the region where monomer structures were formed, which corresponded with the functional domain identified by secondary structure analysis (SAM domain: 255–324 aa) (Figure 4K and **Figure** **5**A), indicating the potential role of SCML1 in transcriptional regulation. We performed ChIP‐Seq to detect potential genes regulated by SCML1 (Figure S4A,B, Supporting Information). Kyoto encyclopedia of genes and genomes (KEGG) analysis of the identified genes showed that the genes regulated by SCML1 played roles in multiple pathways associated with cancer development (Figure 5B), while GO analysis also suggested that genes regulated by SCML1 had important roles in molecular function, cellular components, and biological processes (Figure 5C).

ChIP‐Seq analysis identified the HN polypeptide family as a group of genes potentially regulated by SCML1 (Figure S4C, Supporting Information). HN has a significant protective function in normal cells,^25^, ^26^ but its function and mechanism in lung cancer development is unknown. ChIP‐PCR and ChIP‐qPCR indicated that SCML1 acted on multiple members of the HN polypeptide family, including HN1, HN2, HN3, HN5, HN6, HN8, HN9, and HN10, particularly HN2 and HN8. Furthermore, transcription levels of HN2 and HN8 were significantly decreased in H460 and A549 cells compared with BEAS‐2B cells (Figure 5D–I). circNOL10‐targeting chromatin isolation by RNA purification (CHIRP)‐PCR and CHIRP‐qPCR further showed that H460 cells expressed all members of the HN family, with particularly high expression of HN1, HN2, and HN8 (Figure 5J,K). These findings suggest that circNOL10 can modulate the transcriptional regulatory effect of SCML1 on the HN polypeptide family. Considering the abundances of HN2 and HN8, we selected these two HN members for further experiments, as representatives of the HN family.

We investigated if SCML1 regulated HN transcription directly by SCML1 ChIP‐Seq, and found little difference between HN2 and HN8 sequences; two motifs (motif 1 and motif 2) in SCML1 matched both HN2 and HN8 (Figure 5L,M). We analyzed the different regions of the HN2 and HN8 sequences and designed DNA EMSA probes targeting HN2, HN8, or HN2 plus HN8. We then determined if SCML1 acted directly on HN2 and/or HN8 by EMSA. A super‐shifted band plus SCML1 band were only detected in nuclear extracts (Figure 5N–P), indicating a direct interaction between SCML1 and HN2 or HN8.

We further investigated the transcriptional regulation of HN by SCML1 by detecting HN2 and HN8 expression with qPCR after silencing and overexpressing SCML1 in H460 and A549 cells. Silencing SCML1 resulted in a significant decrease in HN2 and HN8 expression levels, while SCML1 overexpression had the opposite effects (Figure 5Q). HN2 and HN8 expression levels were also reduced by silencing circNOL10 and increased by circNOL10 overexpression (Figure 5R). Given that low expression of circNOL10 inhibited SCML1 (Figure 4O,Q,R), we supposed that circNOL10 promoted the transcriptional regulatory effect of SCML1 on the HN polypeptide family.

### 2.5 circNOL10 Inhibited Lung Cancer Cell Growth by Regulating the HN Polypeptide Family

**Figure 6:**
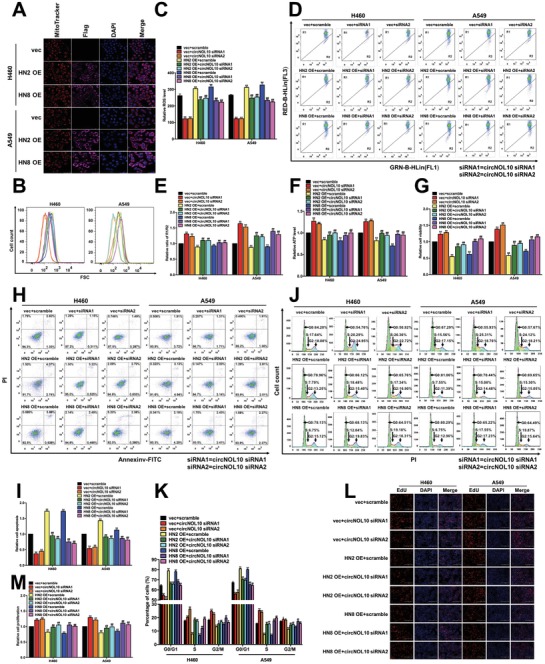
Function of circNOL10 in regulating HN polypeptide family in lung cancer cells. A) Mitochondria were stained using MitoTracker. HN2 and HN8 were labeled with Flag. Nuclei were stained with DAPI. Merge represents an overlay figure. B,C) ROS detection in H460 and A549 cells with FCM. FSC, forward scatter (proportional to cell size). D,E) Mitochondrial membrane potential in H460 and A549 cells detected with FCM. GRN‐B‐HLin (FL1) and RED‐B‐HLin (FL2) represent different channels in FCM assays. F) Relative ATP expression in H460 and A549 cells. G) Cell viability of H460 and A549 cells detected with CCK8 assay. H,I) Apoptosis of H460 and A549 cells detected with FCM. AnnexinV‐FITC and PI were used to indicate apoptosis. J,K) Cell cycle in H460 and A549 cells detected with FCM. Cells stained with PI. Cell count, the number of cells in different cell cycle phases. L,M) Proliferation of H460 and A549 cells detected with EdU assay. Nuclei were stained with DAPI. EdU indicates proliferating cells. C,E,F,G,I,K,M) *n* = 3, mean ± SD; unpaired *t*‐tests used for comparisons between treatment and control groups (vec or scramble), *P < 0.05, **P < 0.01; ANOVA used for comparisons between different treatment groups, ^#^ *P* < 0.05, ^##^ *P* < 0.01.

The HN polypeptide family shows high homology with the mitochondrial gene (MT‐RNR‐like), but its precise origin has not been elucidated.^27^ We studied the subcellular localization of HN using HN2 and HN8 expression vectors with fluorescent flags, using Mito Tracker staining. Confocal laser microscopy indicated that HN2 and HN8 were largely fused, suggesting that they were both mainly distributed in the mitochondria (**Figure** **6**A).

Changes in mitochondrial functions have a profound impact on tumor development.^28^, ^29^ HN2 and HN8 localization in the mitochondrion indicates that the HN family, as well as their regulator circNOL10, may play key roles in affecting mitochondrial function in lung cancer cells. We therefore explored the effects of HN and circNOL10 on mitochondrial function in lung cancer cells (H460 and A549 cells) by overexpressing HN2 and HN8 alone, silencing circNOL10 alone, and silencing circNOL10 combined with overexpressing HN2 and HN8, respectively. Biomarkers indicating mitochondrial function, including reactive oxygen species (ROS) (Figure 6B,C) and mitochondrial membrane potential (MMP), were detected with flow cytometry (Figure 6D,E), and by measuring total adenosine triphosphate (ATP) levels in the cells (Figure 6F). Silencing circNOL10 alone caused a significant decrease in ROS and significant increases in MMP and total ATP levels, while overexpressing HN2 and HN8 alone had the opposite effects. Silencing circNOL10 combined with overexpressing HN2 and HN8 induced a more significant increase in ROS and decreases in MMP and total ATP level compared with overexpressing HN2 and HN8 alone. These results suggest that the HN family (HN2 and HN8) and circNOL10 can both modulate mitochondrial function in lung cancer cells, and circNOL10 may affect mitochondrial function by regulating the HN polypeptide family.

ROS are produced by aerobic metabolism, and can affect tumor cell apoptosis, proliferation, and cell cycle.^30^, ^31^ A decrease in MMP is a marker of the early stage of apoptosis,^32^ while changes in ATP levels are associated with abnormal tumor cell behaviors, including cell viability and apoptosis.^33^, ^34^ We therefore investigated the effects of the HN polypeptide family (HN2, HN8) and circNOL10 on the biological processes and behaviors of H460 and A549 lung cancer cells overexpressing HN2 and HN8 alone, with silencing of circNOL10 alone, or with silencing of circNOL10 combined with overexpressing HN2 and HN8, respectively. We investigated cell viability with CCK8 assay (Figure 6G), apoptosis and cell cycle by flow cytometry (Figure 6H–K and Figure S5A,B, Supporting Information), and cell proliferation by (5‐ethynyl‐2′‐deoxyuridine) (EdU) assay (Figure 6L,M). Silencing circNOL10 alone significantly increased cell viability and proliferation, decreased apoptosis, shortened G0/G1, and prolonged S phase and G2/M phase, while overexpressing HN2 and HN8 alone had the opposite effects. Compared with silencing circNOL10 alone, silencing circNOL10 combined with overexpressing HN2 and HN8 significantly decreased cell viability and proliferation, increased cell apoptosis, prolonged G0/G1 phase, and shortened S and G2/M phases. Compared with overexpressing HN2 and HN8 alone, the combined treatment had the opposite effects to those observed with silencing circNOL10 alone. These results indicated that HN2 and HN8 could inhibit lung cancer cell viability, promote apoptosis, shorten S and G2/M phases, and inhibit cell proliferation, while the inhibitory effect of circNOL10 on lung cancer cell growth may be achieved through regulating the HN polypeptide family.

### 2.6 circNOL10 Altered Multiple Tumor Signaling Pathways by Regulating HN Polypeptide Family

We showed that circNOL10 altered biological processes such as apoptosis, proliferation, and cell cycle in tumor cells through regulating the HN polypeptide family. KEGG pathway analysis (http://www.kegg.jp/kegg/pathway.html) identified 16 tumor biomarkers involved in apoptosis, proliferation, and cell cycle progression. These biomarkers could be divided into two groups: group 1, including the apoptotic biomarkers POLE, MLH3, MSH2, LIG4, p53, and phospho‐p53 (P‐p53), of which POLE, MLH3, MSH2, and LIG4 are associated with DNA damage in apoptotic signal pathways; and group 2, including the proliferation and cell cycle biomarkers CKEK2, phospho‐CHEK2 (P‐CHEK2), CDC25A, phospho‐CDC25A (P‐CDC25A), CCND1, CDK2, Rb, phospho‐Rb (P‐Rb), E2F1, and phospho‐E2F1 (P‐E2F1).

**Figure 7:**
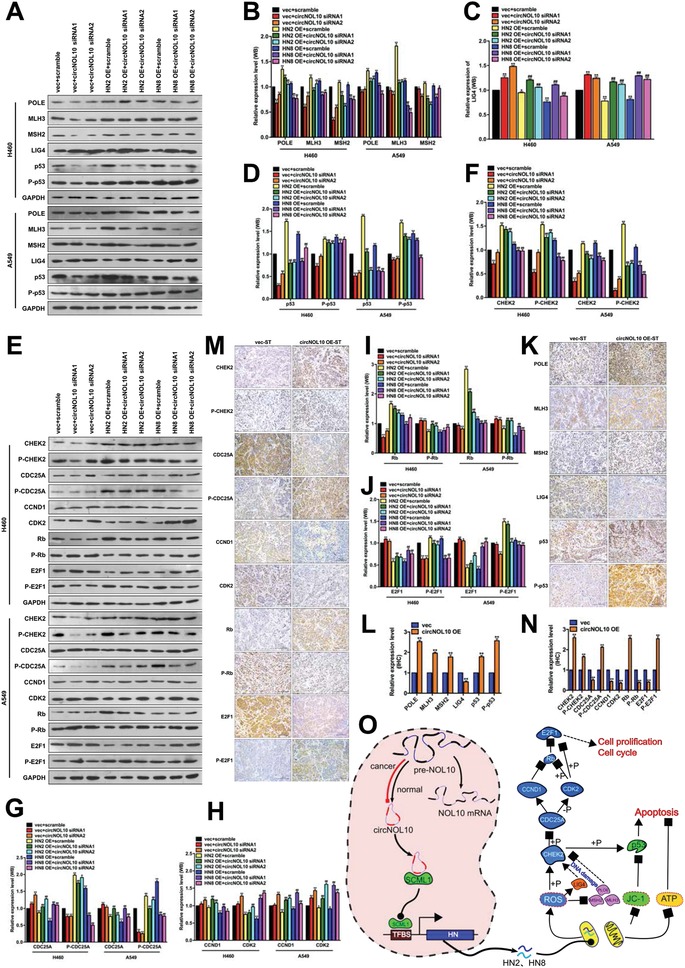
Detection of molecules potentially associated with circNOL10‐regulated inhibitory effect of HN polypeptide family on lung cancer development. A–D) Semi‐quantitative analysis of POLE, MLH3, MSH2, LIG4, p53, and P‐p53 protein expression in H460 and A549 cells, detected with western blot. E–J) Semi‐quantitative analysis of CHEK2, P‐CHEK2, CDC25A, P‐CDC25A, CCND1, CDK2, Rb, P‐Rb, E2F1, and P‐E2F1 protein expression in H460 and A549 cells, detected with western blot. K,L) Quantitative analysis of POLE, MLH3, MSH2, LIG4, p53, and P‐p53 protein expression in subcutaneous tumors in nude mice (circNOL10 overexpression and control) detected by immunohistochemistry. M,N) Quantitative analysis of CHEK2, P‐CHEK2, CDC25A, P‐CDC25A, CCND1, CDK2, Rb, P‐Rb, E2F1, and P‐E2F1 protein expression in subcutaneous tumors in nude mice (circNOL10 overexpression and control), detected by immunohistochemistry. O) Mechanism of circNOL10 in inhibiting lung cancer.

We examined these 16 biomarkers in H460 and A549 cells with western blotting after silencing circNOL10 alone, overexpressing HN2 and HN8 alone, or silencing circNOL10 combined with overexpressing HN2 and HN8 (group 1: **Figure** **7**A–D; group 2: Figure 7E–J). Silencing circNOL10 alone decreased expression levels of ten biomarkers (POLE, MLH3, MSH2, p53, P‐p53, CKEK2, P‐CHEK2, P‐CDC25A, Rb, and P‐E2F1) and increased six (LIG4, CDC25A, CCND1, CDK2, P‐Rb, and E2F1), while overexpressing HN2 and HN8 had the opposite effects. Combined treatment increased the ten biomarkers and decreased the six compared with silencing circNOL10 alone, and had the opposite effects compared with overexpressing HN2 and HN8 alone.

We also investigated the effects of circNOL10 on the 16 biomarkers in vivo. H460 cells transfected with circNOL10 stably overexpressed vector (circNOL10 OE‐ST) or blank vector (vec‐ST) were injected subcutaneously into nude mice to form tumor xenografts and the 16 biomarkers were detected with immunohistochemistry (group 1: Figure 7K,L; group 2: Figure 7M,N). Ten markers (POLE, MLH3, MSH2, p53, P‐p53, CKEK2, P‐CHEK2, P‐CDC25A, Rb, and P‐E2F1) were increased and six (LIG4, CDC25A, CCND1, CDK2, P‐Rb, and E2F1) were decreased in the circNOL10 OE‐ST group compared with the vec‐ST group. Detection of these 16 key molecules with western blot and immunohistochemistry confirmed that circNOL10 inhibited the development of lung cancer by regulating the HN polypeptide family, involving alterations in multiple signaling pathways. The changes in these 16 tumor biomarkers associated with proliferation, cell cycle, and apoptosis were in line with the function of circNOL10 in inhibiting lung cancer development through regulating the HN polypeptide family.

## 3 Discussion

Increasing numbers of circRNAs are being identified and their functions and molecular mechanisms elucidated. circRNAs have been shown to play important roles in various cancers. circRNA MYLK can act as an endogenous competitive RNA to promote the development of bladder cancer through regulating the vascular endothelial growth factor (VEGF)A/VEGF receptor 2 signaling pathway.^35^ circRNACCDC66 was shown to accelerate the proliferation and metastasis of colon cancer,^36^ circLARP4 regulated LARP4 expression by competitively binding to miR‐424‐5p in gastric cancer,^37^ and circLMO7 was involved in the regulation of myogenic differentiation in cattle.^38^ circRNAs have also been shown to regulate the lung cancer phenotype by acting as endogenous competitive RNAs during carcinogenesis and tumor development^39^, ^40^; however, studies on the function and mechanism of circRNAs in lung cancer are still limited. As natural endogenous RNAs, circRNAs have multiple functions associated with their specific structure, and further detailed studies are needed to explore their functions and molecular mechanisms.

In this study, we found that circNOL10 was downregulated in lung cancer cells and in human lung cancer tissues. Analysis of clinical data showed significant differences in circNOL10 expression between lung adenocarcinoma and lung squamous cell carcinoma and in relation to the degree of differentiation. We predicted the function of circNOL10 by bioinformatics and indicated that the circRNA might play key roles in pathways related to the development of lung cancer. We therefore investigated the role of circNOL10 in cells in vitro and in a nude mouse model in vivo, and showed that circNOL10 downregulation significantly inhibited lung cancer. We further explored the reasons for circNOL10 downregulation. RNA methylation is critical for the formation of mature RNAs,^21^ and the formation of circRNAs is also regulated by splicing factors.^18^ Results showed that splicing factor ESRP1 promoted the expression of circNOL10 and NOL10 mRNA. Based on the results, we revealed that both RNA methylation and splicing factors were involved in the downregulation of circNOL10 in lung cancer cells.

circRNAs have diverse functions in cancer; in addition to functioning as endogenous competitive RNAs, some circRNAs encode proteins and are involved in tumors.^41^ However, the role of circRNAs in transcriptional regulation in cancer remains unknown. High ubiquitination of proteins in the cell can promote their degradation, and increased protein expression is sometimes due to inhibition of ubiquitination.^23^, ^24^ The current study showed that circNOL10 enhanced SCML1 expression by inhibiting ubiquitination of the transcription factor SCML1. Furthermore, ChIP‐Seq and molecular experiments revealed that SCML1 regulated transcription of the HN polypeptide family, while circNOL10 affected HN expression by regulating SCML1. The HN polypeptide family has a protective function in normal cells.^26^, ^27^ The HN peptide family has high homology with the mitochondrial genome, but the specific source has not yet been finalized.^27^ HN polypeptide family members (HN2, HN8) are located in mitochondria, and it is therefore important to clarify the effects of HN2 and HN8 on mitochondrial function. We found that two members of the HN family, HN2 and HN8, were located in the mitochondria. The mitochondria are responsible for cellular energy metabolism, and abnormalities in mitochondrial function also contribute to tumor development.^29^, ^30^ We detected biomarkers of mitochondrial function and key molecules involved in proliferation, apoptosis, and cell cycle signaling pathways, and showed that circNOL10 inhibited cell proliferation and cell cycle progression, and promoted apoptosis in lung cancer cells. These effects may be achieved by promoting SCML1 and further regulating transcription of the HN polypeptide family. In this study, we found that circNOL10 regulated multiple tumor signaling pathways in the regulation of the HN polypeptide family, by detecting a variety of tumor signaling pathway molecules.

The results of this study provide new insights into the role of circRNAs in lung cancer, and provide evidence for the molecular mechanisms whereby circRNAs participate in transcriptional regulation. Furthermore, we showed that circNOL10 inhibited lung cancer by enhancing transcriptional regulation of the HN polypeptide family by SCML1. These findings further our understanding of lung cancer and may help to identify new diagnostic and therapeutic targets. This study revealed that circNOL10 was significantly downregulated in lung cancer and was involved in lung cancer inhibition. circNOL10 downregulation in lung cancer cells was co‐regulated by Pre‐NOL10 methylation and the splicing factor ESRP1. Furthermore, circNOL10 promoted expression of the transcription factor SCML1 and further induced transcription of the HN polypeptide family in lung cancer. Members of the HN polypeptide family can be transferred into mitochondria to affect mitochondrial function and key biological processes such as apoptosis, proliferation, and cell cycle progression in lung cancer cells, which may be mediated by multiple signaling pathways (Figure 7O and Figure S, Supporting Information).

## 4 Experimental Section

*Cell and Animal Experiments and Human Tissue Samples*: The cells used in this study were all cultured as recommended by the American Type Culture Collection. All cell lines were authenticated by short tandem repeat profiling. All the animal experiments in this study were approved by the Experimental Animal Ethics Committee of Guangzhou Medical University. The experimental animals were maintained under specific‐pathogen‐free conditions at the Experimental Animal Center of Guangzhou Medical University, and fed in accordance with the rules of the center. The BALB/c‐nu/nu mice used in this study were provided by the Guangdong Medical Laboratory Animal Center. Human tissue samples were provided by the tissue bank of the Institute of Chemical Carcinogenesis of Guangzhou Medical University, from individuals from Guangzhou City. Informed consent was obtained from all patients before sample collection. This study was approved by the Medical Ethics Committee of Guangzhou Medical University.

*High‐Throughput Sequencing and Protein MS*: High‐throughput ChIP‐Seq was performed with RAP‐MS and pull‐down MS. Sequencing results and protein MS results are listed in the Supporting Information.

*Statistical Analysis*: The experimental data were analyzed using SPSS 17.0 software (SPSS Inc., Chicago, IL, USA). The data were expressed as mean ± SD, based on *n* = 3. The data were analyzed using paired or unpaired *t*‐tests or ANOVA, as appropriate. A *P* value of <0.05 was considered statically significant.

“Detailed methods are described in the Supporting Information.”

## Conflict of Interest

The authors declare no conflict of interest.

## Supporting information

Supplementary
