# Supplementary material for: Circular RNA circNOL10 Inhibits Lung Cancer Development by Promoting SCLM1‐Mediated Transcriptional Regulation of the Humanin Polypeptide Family
Source: Adv Sci (Weinh). 2018 Nov 16;6(2):1800654. doi: 10.1002/advs.201800654 (PMC6343086; doi:10.1002/advs.201800654)
Supplement: Supplementary file 1 — Supplementary [file ADVS-6-1800654-s001.pdf]

Copyright WILEY-VCH Verlag GmbH & Co. KGaA, 69469 Weinheim, Germany, 2018.

ADVANCED SCIENCE
Open Access

Supporting Information

for *Adv. Sci.,* DOI: 10.1002/advs.201800654

Circular RNA circNOL10 Inhibits Lung Cancer Development by Promoting SCLM1-Mediated Transcriptional Regulation of the Humanin Polypeptide Family

*Aruo Nan, Lijian Chen, Nan Zhang, Yangyang Jia, Xin Li, Hanyu Zhou, Yihui Ling, Zhishan Wang, Chengfeng Yang, Sijin Liu, and Yiguo Jiang**

Supplementary Materials

Supplemental Figures

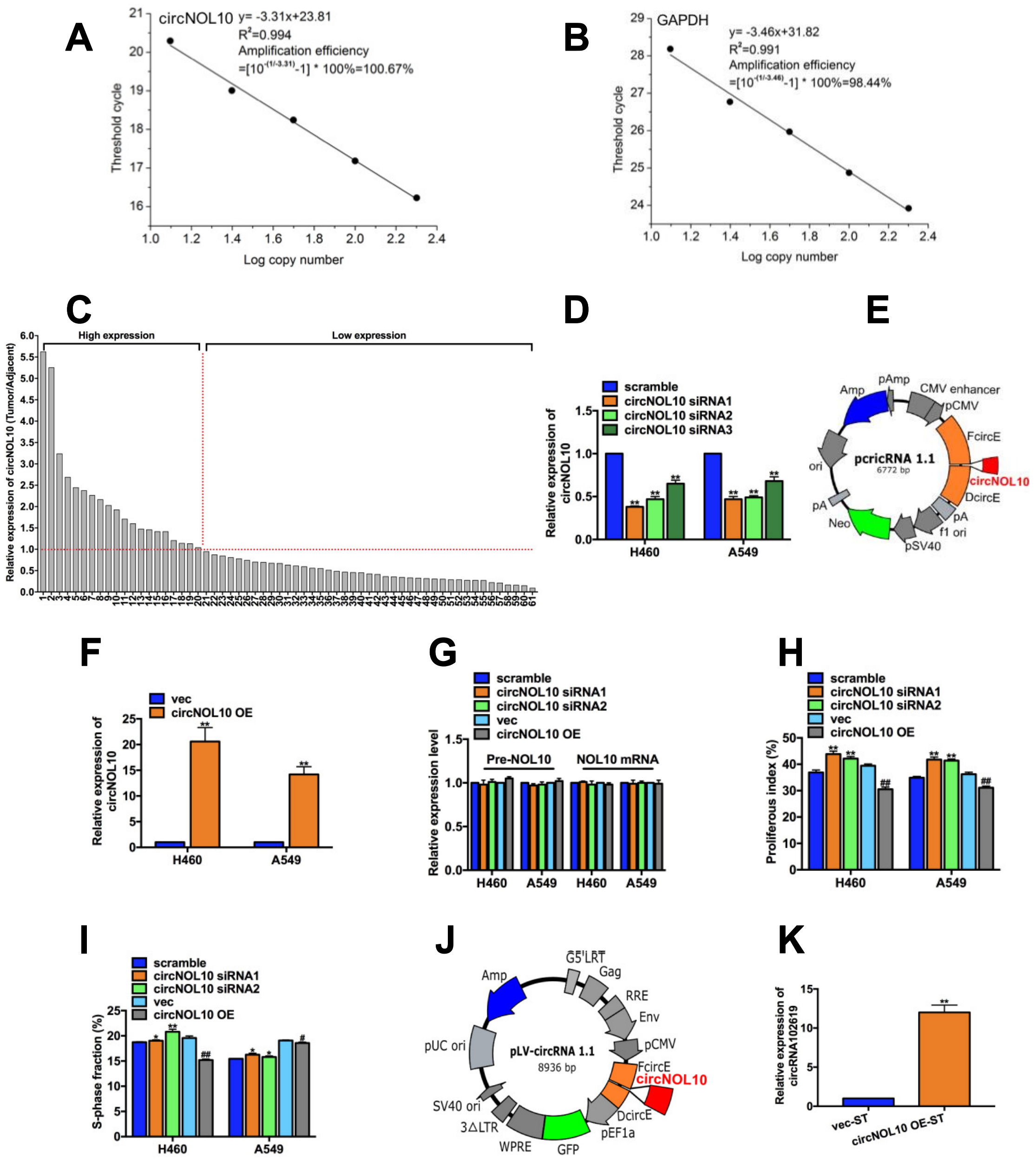

Supplemental Figure 1 (related to Figures 1 and 2). Significant inhibitory effect of circNOL10 on lung cancer development in vitro and in vivo.

(A and B) Amplification efficiencies of the primers for the gene of interest (circNOL10) and reference gene (GAPDH) were 100.67% and 98.44%, respectively.

(C) circNOL10 mRNA expression in 61 cases of human lung cancer and matched paracancerous tissues, determined with qPCR. Twenty cases showed high circNOL10 expression and 41 cases showed low expression.

(D) Silencing efficiency of transient transfection with three siRNAs in H460 and

A549 cells; n = 3, mean ± SD, unpaired t-test, **P<0.01.

(E) circNOL10-specific overexpression vector for transient transfection.

(F) Overexpression efficiency of transient transfection with circNOL10 overexpression vector in H460 and A549 cells; n = 3, mean ± SD, unpaired t-test, **P<0.01.

(G) Expression of pre-NOL10 and NOL10 mRNAs in H460 and A549 cells after silencing or overexpressing circNOL10, detected with qPCR; n = 3, mean ± SD, unpaired t-test, not significant.

(H, I) Cell proliferation index and proportion of S-phase cells detected with flow cytometry (FCM); n = 3, mean ± SD, unpaired t-test, **P<0.01. Related to Figure 1L.

(J) Green fluorescent protein-labeled circNOL10 specific overexpression vector for stable transfection.

(K) Efficiency of stable transfection with circNOL10 overexpression vector in H460 cells. n = 3, mean ± SD, unpaired t-test, **P<0.01.

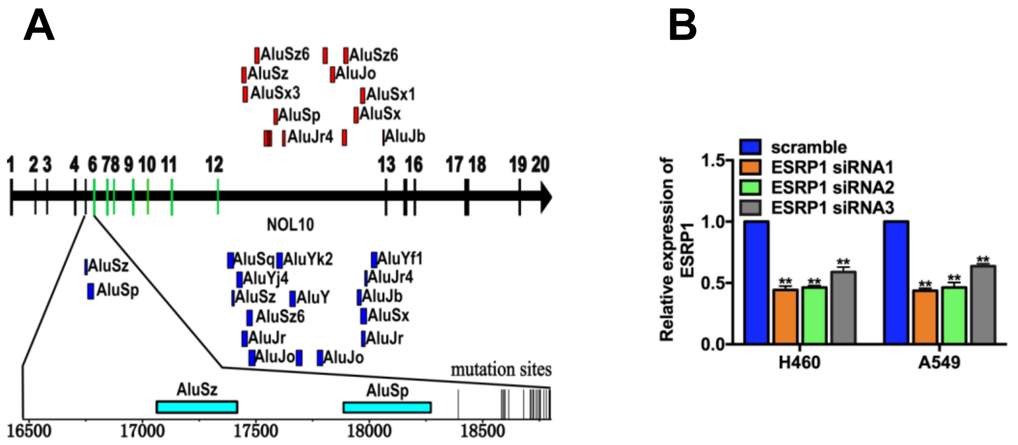

Supplemental Figure 2 (related to Figure 3). Low-expression mechanism of circNOL10 in lung cancer.

(A) Sequencing of flanking intron region in circNOL10. There were few mutation sites and the detected mutation sites were not in the circNOL10-associated circularization region.

(B) Silencing efficiency of transient transfection with three ESRP1 siRNAs in H460 and A549 cells; n = 3, mean ± SD, unpaired t-test, **P<0.01.

A

Protein	Annotation
MYH9_HUMAN	Myosin-9 OS=Homo sapiens GN=MYH9 PE=1 SV=4
ACTB_HUMAN	Actin, cytoplasmic 1 OS=Homo sapiens GN=ACTB PE=1 SV=1
ACTC_HUMAN	Actin, alpha cardiac muscle 1 OS=Homo sapiens GN=ACTC1 PE=1 SV=1
POTEE_HUMAN	POTE ankyrin domain family member E OS=Homo sapiens GN=POTEE PE=1 SV=3
ACTBL_HUMAN	Beta-actin-like protein 2 OS=Homo sapiens GN=ACTBL2 PE=1 SV=2
MYL6_HUMAN	Myosin light polypeptide 6 OS=Homo sapiens GN=MYL6 PE=1 SV=2
MYH10_HUMAN	Myosin-10 OS=Homo sapiens GN=MYH10 PE=1 SV=3
CPSF5_HUMAN	Cleavage and polyadenylation specificity factor subunit 5 OS=Homo sapiens GN=NUDT21 PE=1 SV=1
TPM1_HUMAN	Tropomyosin alpha-1 chain OS=Homo sapiens GN=TPM1 PE=1 SV=2
TBB5_HUMAN	Tubulin beta chain OS=Homo sapiens GN=TUBB PE=1 SV=2
ML12A_HUMAN	Myosin regulatory light chain 12A OS=Homo sapiens GN=MYL12A PE=1 SV=2
TPM3_HUMAN	Tropomyosin alpha-3 chain OS=Homo sapiens GN=TPM3 PE=1 SV=2
SF3B3_HUMAN	Splicing factor 3B subunit 3 OS=Homo sapiens GN=SF3B3 PE=1 SV=4
K2C1_HUMAN	Keratin, type II cytoskeletal 1 OS=Homo sapiens GN=KRT1 PE=1 SV=6
LCA5L_HUMAN	Lebercilin-like protein OS=Homo sapiens GN=LCA5L PE=1 SV=1
XKR4_HUMAN	XK-related protein 4 OS=Homo sapiens GN=XKR4 PE=2 SV=1
SF3B1_HUMAN	Splicing factor 3B subunit 1 OS=Homo sapiens GN=SF3B1 PE=1 SV=3
SCML1_HUMAN	Sex comb on midleg-like protein 1 OS=Homo sapiens GN=SCML1 PE=1 SV=2

B

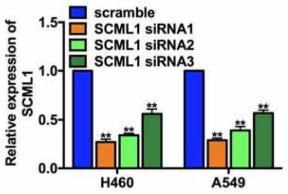

C

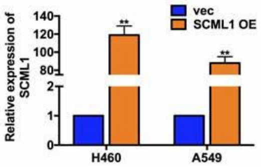

D

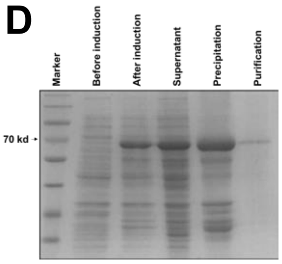

E

Peptide	Position	Score	Threshold
KIDVIRRKVSKIQRF	85	0.36	0.3
VIRRKVSKIQRFHAR	88	0.84	0.3
NHKRYGYKKHSYRLV	107	0.65	0.3
HKRYGYKKHSYRLVK	108	0.99	0.3
KHSYRLVKKLKLQKM	115	1.02	0.3
HSYRLVKKLKLQKMK	116	0.51	0.3
YRLVKKLKLQKMKKN	118	1.06	0.3
VKKLKLQKMKKNEVY	121	1.3	0.3
KLKLQKMKKNEVYET	123	0.7	0.3
LKLQKMKKNEVYETF	124	0.61	0.3
EAVVLFLKQTDPLAL	268	1.6	0.3
RSHEIDGKALLLLTS	290	0.63	0.3
LTSDVLLKHLGVKLG	302	0.37	0.3
LLKHLGVKLGTAVKL	307	1.72	0.3
IDRLKQGKCFEN***	325	1.1	0.3

F

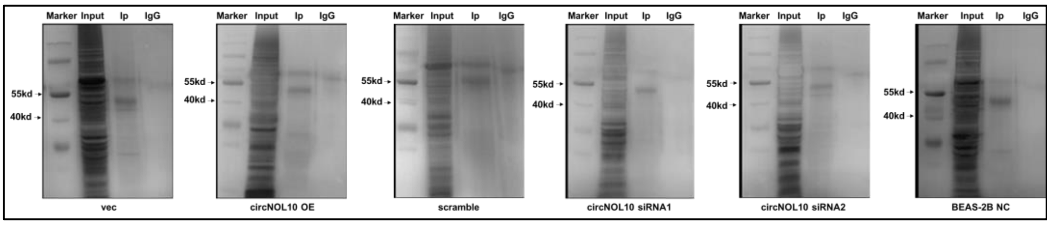

Supplemental Figure 3 (related to Figure 4). Mechanism of circNOL10 in regulating transcription factor SCML1.

(A) Proteins with a relatively high abundance in circNOL10 pull-down-MS included SCML1.

(B) Silencing efficiency of transient transfection with three SCML1 siRNAs in H460 and A549 cells; n = 3, mean ± SD, unpaired t-test, **P<0.01.

(C) Efficiency of transient transfection with SCML1 overexpression vector in H460 and A549 cells; n = 3, mean ± SD, unpaired t-test, **P<0.01.

(D) Silver staining of prokaryotically expressed and purified SCML1 protein, used for the detection of RNA EMSA.

(E) SCML1 ubiquitination sites (red indicates predicted ubiquitination sites).

(F) Silver staining of proteins obtained by SCML1 co-immunoprecipitation. circNOL10 was silenced or overexpressed in H460 cells and BEAS-2B cells were untreated.

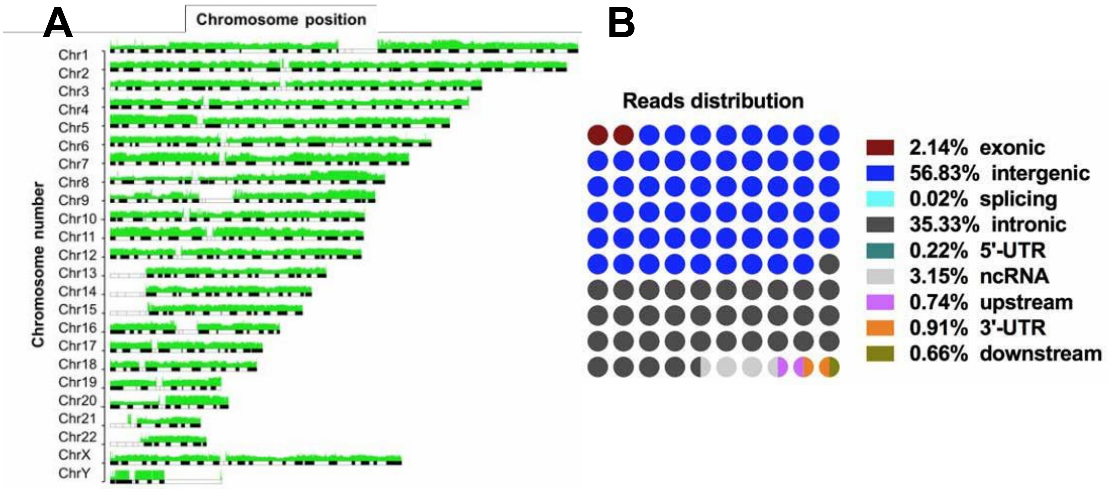

C

PeakID	Chr	Start	End	Annotation	Nearest PromoterID	Nearest Ensemble	Gene Name	Gene Alias	Gene Description	Gene Type
MACS_peak_1902	chr17	22022697	22022915	5' UTR (NM_001190452, exon 1 of 1)	NM_001190452	ENSG00000256618	MTRNR2L1	HN1	MT-RNR2-like 1	protein-coding
MACS_peak_1910	chr17	22260254	22262998	Intergenic	NM_001190452	ENSG00000256618	MTRNR2L1	HN1	MT-RNR2-like 1	protein-coding
MACS_peak_1909	chr17	22244427	22246914	Intergenic	NM_001190452	ENSG00000256618	MTRNR2L1	HN1	MT-RNR2-like 1	protein-coding
MACS_peak_1900	chr17	22020682	22020775	Intergenic	NM_001190452	ENSG00000256618	MTRNR2L1	HN1	MT-RNR2-like 1	protein-coding
MACS_peak_1905	chr17	22027639	22027794	Intergenic	NM_001190452	ENSG00000256618	MTRNR2L1	HN1	MT-RNR2-like 1	protein-coding
MACS_peak_1901	chr17	22022121	22022299	promoter-TSS (NM_001190452)	NM_001190452	ENSG00000256618	MTRNR2L1	HN1	MT-RNR2-like 1	protein-coding
MACS_peak_3888	chr5	79946590	79946774	5' UTR (NM_001190470, exon 1 of 1)	NM_001190470	ENSG00000271043	MTRNR2L2	HN2	MT-RNR2-like 2	protein-coding
MACS_peak_3887	chr5	79946243	79946535	5' UTR (NM_001190470, exon 1 of 1)	NM_001190470	ENSG00000271043	MTRNR2L2	HN2	MT-RNR2-like 2	protein-coding
MACS_peak_3886	chr5	79945910	79946073	5' UTR (NM_001190470, exon 1 of 1)	NM_001190470	ENSG00000271043	MTRNR2L2	HN2	MT-RNR2-like 2	protein-coding
MACS_peak_3890	chr5	79947144	79947501	promoter-TSS (NM_001190470)	NM_001190470	ENSG00000271043	MTRNR2L2	HN2	MT-RNR2-like 2	protein-coding
MACS_peak_3889	chr5	79946917	79947108	promoter-TSS (NM_001190470)	NM_001190470	ENSG00000271043	MTRNR2L2	HN2	MT-RNR2-like 2	protein-coding
MACS_peak_2857	chr20	55933986	55934307	5' UTR (NM_001190472, exon 1 of 1)	NM_001190472	ENSG00000256222	MTRNR2L3	HN3	MT-RNR2-like 3	protein-coding
MACS_peak_594	chr10	57359628	57359806	exon (NM_001190478, exon 1 of 1)	NM_001190478	ENSG00000249860	MTRNR2L5	HN5	MT-RNR2-like 5	protein-coding
MACS_peak_593	chr10	57357863	57357932	promoter-TSS (NM_001190478)	NM_001190478	ENSG00000249860	MTRNR2L5	HN5	MT-RNR2-like 5	protein-coding
MACS_peak_4719	chr7	142375225	142375458	3' UTR (NM_001190487, exon 1 of 1)	NM_001190487	ENSG00000270672	MTRNR2L6	HN6	MT-RNR2-like 6	protein-coding
MACS_peak_737	chr11	10530669	10531352	promoter-TSS (NM_001190702)	NM_001190702	ENSG00000255823	MTRNR2L8	HN8	MT-RNR2-like 8	protein-coding
MACS_peak_738	chr11	10531472	10531605	promoter-TSS (NM_001190702)	NM_001190702	ENSG00000255823	MTRNR2L8	HN8	MT-RNR2-like 8	protein-coding
MACS_peak_736	chr11	10530261	10530539	promoter-TSS (NR_039705)	NM_001190702	ENSG00000255823	MTRNR2L8	HN8	MT-RNR2-like 8	protein-coding
MACS_peak_4143	chr6	62284042	62284224	5' UTR (NM_001190706, exon 1 of 1)	NM_001190706	ENSG00000255633	MTRNR2L9	HN9	MT-RNR2-like 9 (pseudogene)	protein-coding
MACS_peak_4141	chr6	61913273	61913625	Intergenic	NM_001190706	ENSG00000255633	MTRNR2L9	HN9	MT-RNR2-like 9 (pseudogene)	protein-coding
MACS_peak_4137	chr6	61905130	61905466	Intergenic	NM_001190706	ENSG00000255633	MTRNR2L9	HN9	MT-RNR2-like 9 (pseudogene)	protein-coding
MACS_peak_4140	chr6	61912894	61913137	Intergenic	NM_001190706	ENSG00000255633	MTRNR2L9	HN9	MT-RNR2-like 9 (pseudogene)	protein-coding
MACS_peak_4139	chr6	61911289	61911593	Intergenic	NM_001190706	ENSG00000255633	MTRNR2L9	HN9	MT-RNR2-like 9 (pseudogene)	protein-coding
MACS_peak_4142	chr6	61914100	61914485	Intergenic	NM_001190706	ENSG00000255633	MTRNR2L9	HN9	MT-RNR2-like 9 (pseudogene)	protein-coding
MACS_peak_4135	chr6	61902165	61902270	Intergenic	NM_001190706	ENSG00000255633	MTRNR2L9	HN9	MT-RNR2-like 9 (pseudogene)	protein-coding
MACS_peak_5355	chrX	55208020	55208207	3' UTR (NM_001190708, exon 1 of 1)	NM_001190708	ENSG00000256045	MTRNR2L10	HN10	MT-RNR2-like 10	protein-coding
MACS_peak_5354	chrX	55204838	55204922	Intergenic	NM_001190708	ENSG00000256045	MTRNR2L10	HN10	MT-RNR2-like 10	protein-coding
MACS_peak_5357	chrX	55209824	55209997	promoter-TSS (NM_001190708)	NM_001190708	ENSG00000256045	MTRNR2L10	HN10	MT-RNR2-like 10	protein-coding

Supplemental Figure 4 (related to Figure 5). Mechanism of SCML1 in regulating HN peptide family.

(A) Chromosomal distribution of SCML1 ChIP-Seq reads. Horizontal axis represents chromosome position, vertical axis represents chromosome number, and green curve represents read abundance (the higher the peak, the more reads in this position).

(B) Distribution of SCML1 ChIP-Seq reads in different genomic regions (exonic, intergenic, splicing, intronic, 5'-untranslated region (UTR), non-coding RNA (ncRNA), upstream, 3'-UTR and downstream).

(C) Related gene information for the HN peptide family identified with SCML1 ChIP-Seq.

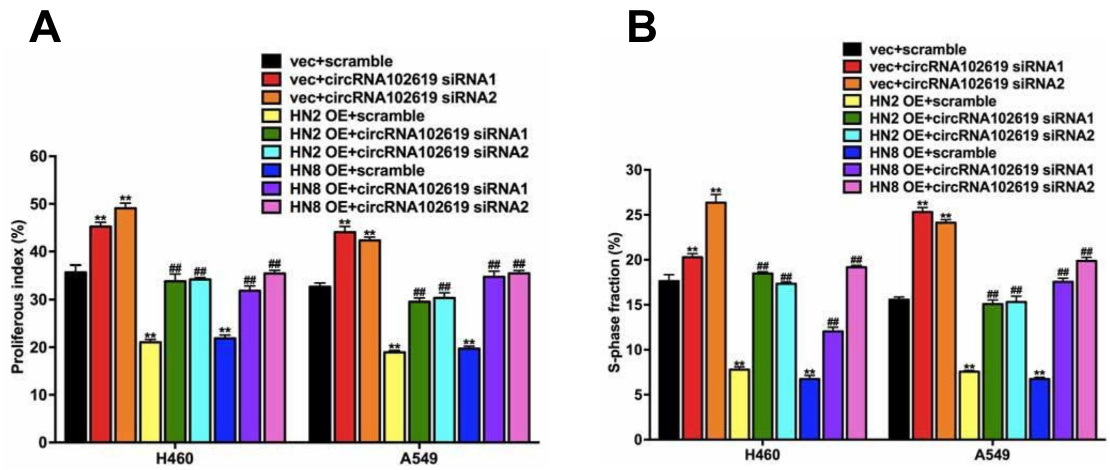

Supplemental Figure 5 (related to Figure 6). Function of circNOL10 in lung cancer cells through regulating HN peptide family.

(A, B) Cell proliferation index and proportion of S-phase cells by FCM (n = 3, mean ± SD). Unpaired t-tests were used to compare different treatment groups and control groups (vec+scramble), **P<0.01. ANOVA was used to compare different treatment groups, ## P< 0.01.

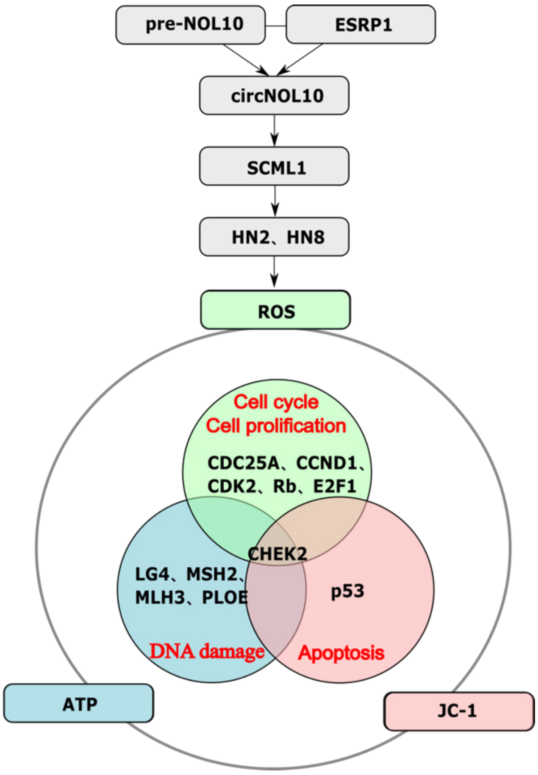

Supplemental Figure 6. Schematic figure of the regulatory mechanism of circNOL10.

Supplemental Table 1. High-throughput circRNA data

probeID	Fold Change	FC (abs)	Log Fold change	Regulation	circRNA	Alias
ASCRP000002	-2.5813426	2.5813426	-1.3681216	down	hsa_circRNA_000010	hsa_circ_0000263
ASCRP000004	-2.6014376	2.6014376	-1.3793091	down	hsa_circRNA_000031	hsa_circ_0000009
ASCRP000006	-2.6118562	2.6118562	-1.3850755	down	hsa_circRNA_000042	hsa_circ_0000036
ASCRP000007	-2.3122878	2.3122878	-1.2093209	down	hsa_circRNA_000046	hsa_circ_0000059
ASCRP000010	-2.0616887	2.0616887	-1.0438265	down	hsa_circRNA_000094	hsa_circ_0000247
ASCRP000019	-4.4177762	4.4177762	-2.1433203	down	hsa_circRNA_000178	hsa_circ_0000549
ASCRP000022	-3.0940504	3.0940504	-1.6294967	down	hsa_circRNA_000200	hsa_circ_0000670
ASCRP000026	-3.3699576	3.3699576	-1.7527305	down	hsa_circRNA_000250	hsa_circ_0000848
ASCRP000027	-2.2659371	2.2659371	-1.1801078	down	hsa_circRNA_000274	hsa_circ_0000919
ASCRP000035	-2.9793332	2.9793332	-1.5749895	down	hsa_circRNA_000329	hsa_circ_0001138
ASCRP000045	-2.9396315	2.9396315	-1.5556353	down	hsa_circRNA_000424	hsa_circ_0001549
ASCRP000074	-5.4808939	5.4808939	-2.4544112	down	hsa_circRNA_000596	hsa_circ_0000661
ASCRP000078	-3.4605588	3.4605588	-1.791005	down	hsa_circRNA_000618	hsa_circ_0000708
ASCRP000084	-2.0844496	2.0844496	-1.0596665	down	hsa_circRNA_000626	hsa_circ_0000755
ASCRP000085	-4.8104975	4.8104975	-2.2661861	down	hsa_circRNA_000629	hsa_circ_0000775
ASCRP000086	-2.2092061	2.2092061	-1.143528	down	hsa_circRNA_000638	hsa_circ_0000822
ASCRP000096	-2.1228304	2.1228304	-1.0859891	down	hsa_circRNA_000676	hsa_circ_0001234
ASCRP000098	-45.33666	45.3366606	-5.5026062	down	hsa_circRNA_000684	hsa_circ_0001013
ASCRP000140	-8.8101937	8.8101937	-3.1391737	down	hsa_circRNA_000864	hsa_circ_0000662
ASCRP000144	-11.029611	11.0296118	-3.4633101	down	hsa_circRNA_000881	hsa_circ_0000788
ASCRP000150	-7.9903767	7.9903767	-2.9982635	down	hsa_circRNA_000911	hsa_circ_0001184
ASCRP000161	-7.1779314	7.1779314	-2.8435681	down	hsa_circRNA_000950	hsa_circ_0001525
ASCRP000167	-3.0573031	3.0573031	-1.6122596	down	hsa_circRNA_000963	hsa_circ_0001644
ASCRP000172	-4.1667918	4.1667918	-2.058937	down	hsa_circRNA_000993	hsa_circ_0001887
ASCRP000174	-2.0181414	2.0181414	-1.0130272	down	hsa_circRNA_000997	hsa_circ_0001845
ASCRP000179	-2.4699397	2.4699397	-1.3044758	down	hsa_circRNA_001036	hsa_circ_0000436
ASCRP000199	-4.2371127	4.2371127	-2.0830815	down	hsa_circRNA_001096	hsa_circ_0000866
ASCRP000231	-2.722984	2.722984	-1.4451885	down	hsa_circRNA_001225	hsa_circ_0000305
ASCRP000253	-7.1985053	7.1985053	-2.8476974	down	hsa_circRNA_001350	hsa_circ_0000253

ASCRP000254	-2.0309892	2.0309892	-1.0221826	down	hsa_circRNA_001355	hsa_circ_0000312
ASCRP000256	-2.3592331	2.3592331	-1.238318	down	hsa_circRNA_001363	hsa_circ_0000172
ASCRP000263	-2.9059766	2.9059766	-1.5390231	down	hsa_circRNA_001389	hsa_circ_0000729
ASCRP000267	-2.9788281	2.9788281	-1.5747449	down	hsa_circRNA_001405	hsa_circ_0001167
ASCRP000303	-3.4304586	3.4304586	-1.7784014	down	hsa_circRNA_001594	hsa_circ_0001343
ASCRP000308	-3.4192764	3.4192764	-1.7736911	down	hsa_circRNA_001622	hsa_circ_0000144
ASCRP000310	-15.452945	15.4529451	-3.9498099	down	hsa_circRNA_001653	hsa_circ_0001568
ASCRP000311	-8.3350025	8.3350025	-3.0591826	down	hsa_circRNA_001654	hsa_circ_0001605
ASCRP000384	-2.206182	2.206182	-1.1415518	down	hsa_circRNA_002143	hsa_circ_0001907
ASCRP000414	-4.3617806	4.3617806	-2.1249172	down	hsa_circRNA_100024	hsa_circ_0009135
ASCRP000434	-10.626184	10.6261847	-3.4095518	down	hsa_circRNA_100044	hsa_circ_0009654
ASCRP000459	-5.1948437	5.1948437	-2.3770803	down	hsa_circRNA_100069	hsa_circ_0002733
ASCRP000462	-2.5503107	2.5503107	-1.350673	down	hsa_circRNA_100072	hsa_circ_0005562
ASCRP000463	-2.5331061	2.5331061	-1.3409075	down	hsa_circRNA_100073	hsa_circ_0010358
ASCRP000557	-2.4018306	2.4018306	-1.2641344	down	hsa_circRNA_100168	hsa_circ_0004660
ASCRP000570	-3.8293529	3.8293529	-1.9371006	down	hsa_circRNA_100181	hsa_circ_0011929
ASCRP000575	-2.1382768	2.1382768	-1.0964486	down	hsa_circRNA_100186	hsa_circ_0000063
ASCRP000591	-4.8075969	4.8075969	-2.2653159	down	hsa_circRNA_100202	hsa_circ_0012185
ASCRP000611	-4.4652407	4.4652407	-2.158738	down	hsa_circRNA_100223	hsa_circ_0012417
ASCRP000618	-2.0632634	2.0632634	-1.044928	down	hsa_circRNA_100230	hsa_circ_0009076
ASCRP000660	-2.3442028	2.3442028	-1.2290974	down	hsa_circRNA_100272	hsa_circ_0004463
ASCRP000706	-2.542756	2.542756	-1.346393	down	hsa_circRNA_100319	hsa_circ_0013912
ASCRP000714	-2.9656454	2.9656454	-1.5683461	down	hsa_circRNA_100329	hsa_circ_0006352
ASCRP000746	-2.3741001	2.3741001	-1.2473808	down	hsa_circRNA_100361	hsa_circ_0008339
ASCRP000776	-2.6384245	2.6384245	-1.3996767	down	hsa_circRNA_100391	hsa_circ_0015243
ASCRP000779	-3.8329609	3.8329609	-1.9384593	down	hsa_circRNA_100395	hsa_circ_0015278
ASCRP000803	-4.9425512	4.9425512	-2.3052559	down	hsa_circRNA_100420	hsa_circ_0015787
ASCRP000816	-3.1270904	3.1270904	-1.6448209	down	hsa_circRNA_100433	hsa_circ_0003600
ASCRP000821	-2.4335872	2.4335872	-1.2830845	down	hsa_circRNA_100438	hsa_circ_0006827
ASCRP000830	-2.8121844	2.8121844	-1.4916912	down	hsa_circRNA_100447	hsa_circ_0016418
ASCRP000836	-27.52202	27.5220205	-4.7825145	down	hsa_circRNA_100453	hsa_circ_0008659
ASCRP000880	-12.200351	12.2003516	-3.6088508	down	hsa_circRNA_100498	hsa_circ_0017109
ASCRP000906	-2.5368909	2.5368909	-1.3430615	down	hsa_circRNA_100525	hsa_circ_0017443

ASCRP000929	-2.8030209	2.8030209	-1.4869825	down	hsa_circRNA_100549	hsa_circ_0004630
ASCRP000949	-2.1428163	2.1428163	-1.0995082	down	hsa_circRNA_100570	hsa_circ_0001963
ASCRP000981	-2.4763365	2.4763365	-1.3082074	down	hsa_circRNA_100606	hsa_circ_0018529
ASCRP000995	-2.0893639	2.0893639	-1.0630638	down	hsa_circRNA_100621	hsa_circ_0018814
ASCRP001030	-2.4258061	2.4258061	-1.2784643	down	hsa_circRNA_100656	hsa_circ_0004896
ASCRP001070	-2.0956982	2.0956982	-1.067431	down	hsa_circRNA_100696	hsa_circ_0008896
ASCRP001087	-2.5263444	2.5263444	-1.3370513	down	hsa_circRNA_100713	hsa_circ_0008915
ASCRP001088	-2.1252266	2.1252266	-1.0876167	down	hsa_circRNA_100714	hsa_circ_0020336
ASCRP001093	-2.9067863	2.9067863	-1.539425	down	hsa_circRNA_100719	hsa_circ_0020390
ASCRP001100	-3.3442055	3.3442055	-1.7416635	down	hsa_circRNA_100726	hsa_circ_0002456
ASCRP001124	-2.3015782	2.3015782	-1.2026234	down	hsa_circRNA_100750	hsa_circ_0020929
ASCRP001133	-3.9111276	3.9111276	-1.9675846	down	hsa_circRNA_100759	hsa_circ_0004099
ASCRP001164	-2.394036	2.394036	-1.2594449	down	hsa_circRNA_100790	hsa_circ_0000288
ASCRP001176	-2.4615486	2.4615486	-1.2995662	down	hsa_circRNA_100802	hsa_circ_0009018
ASCRP001195	-2.8423144	2.8423144	-1.5070661	down	hsa_circRNA_100821	hsa_circ_0005204
ASCRP001219	-4.4338576	4.4338576	-2.1485624	down	hsa_circRNA_100845	hsa_circ_0022614
ASCRP001223	-2.704715	2.704715	-1.4354766	down	hsa_circRNA_100849	hsa_circ_0004291
ASCRP001236	-3.7707498	3.7707498	-1.9148514	down	hsa_circRNA_100862	hsa_circ_0023182
ASCRP001254	-2.3827077	2.3827077	-1.252602	down	hsa_circRNA_100881	hsa_circ_0007722
ASCRP001256	-3.5627105	3.5627105	-1.8329753	down	hsa_circRNA_100883	hsa_circ_0005918
ASCRP001261	-2.153045	2.153045	-1.1063785	down	hsa_circRNA_100888	hsa_circ_0023642
ASCRP001277	-2.7975538	2.7975538	-1.4841659	down	hsa_circRNA_100904	hsa_circ_0007767
ASCRP001343	-2.0069672	2.0069672	-1.005017	down	hsa_circRNA_100972	hsa_circ_0024609
ASCRP001375	-3.5788038	3.5788038	-1.8394774	down	hsa_circRNA_101004	hsa_circ_0000375
ASCRP001410	-3.0259203	3.0259203	-1.597374	down	hsa_circRNA_101040	hsa_circ_0025822
ASCRP001423	-2.2258901	2.2258901	-1.1543823	down	hsa_circRNA_101055	hsa_circ_0026218
ASCRP001426	-2.0284957	2.0284957	-1.0204103	down	hsa_circRNA_101058	hsa_circ_0026285
ASCRP001505	-12.895423	12.8954236	-3.6887873	down	hsa_circRNA_101139	hsa_circ_0028173
ASCRP001527	-9.8196832	9.8196832	-3.2956765	down	hsa_circRNA_101164	hsa_circ_0028602
ASCRP001537	-2.0031553	2.0031553	-1.0022743	down	hsa_circRNA_101174	hsa_circ_0028864
ASCRP001550	-2.1650518	2.1650518	-1.1144016	down	hsa_circRNA_101187	hsa_circ_0006030
ASCRP001570	-3.3978577	3.3978577	-1.7646255	down	hsa_circRNA_101207	hsa_circ_0006689
ASCRP001576	-18.3349	18.3349003	-4.1965205	down	hsa_circRNA_101213	hsa_circ_0029426

ASCRP001582	-8.6522504	8.6522504	-3.1130754	down	hsa_circRNA_101219	hsa_circ_0029584
ASCRP001588	-2.060739	2.060739	-1.0431618	down	hsa_circRNA_101225	hsa_circ_0029633
ASCRP001589	-2.7558171	2.7558171	-1.4624801	down	hsa_circRNA_101226	hsa_circ_0029634
ASCRP001611	-2.367029	2.367029	-1.2430774	down	hsa_circRNA_101248	hsa_circ_0029976
ASCRP001620	-2.3118206	2.3118206	-1.2090294	down	hsa_circRNA_101258	hsa_circ_0004711
ASCRP001639	-4.0340843	4.0340843	-2.0122412	down	hsa_circRNA_101278	hsa_circ_0004217
ASCRP001643	-2.9654851	2.9654851	-1.5682681	down	hsa_circRNA_101282	hsa_circ_0030569
ASCRP001650	-2.0394544	2.0394544	-1.0281832	down	hsa_circRNA_101290	hsa_circ_0030724
ASCRP001664	-2.8428521	2.8428521	-1.5073391	down	hsa_circRNA_101305	hsa_circ_0002807
ASCRP001679	-4.1363664	4.1363664	-2.048364	down	hsa_circRNA_101320	hsa_circ_0031250
ASCRP001693	-2.510809	2.510809	-1.3281523	down	hsa_circRNA_101334	hsa_circ_0002301
ASCRP001732	-3.5999228	3.5999228	-1.847966	down	hsa_circRNA_101373	hsa_circ_0005139
ASCRP001740	-3.7442496	3.7442496	-1.9046766	down	hsa_circRNA_101381	hsa_circ_0004781
ASCRP001775	-2.8150429	2.8150429	-1.4931569	down	hsa_circRNA_101416	hsa_circ_0032821
ASCRP001842	-45.260254	45.2602544	-5.5001728	down	hsa_circRNA_101491	hsa_circ_0034762
ASCRP001876	-3.2830178	3.2830178	-1.7150226	down	hsa_circRNA_101525	hsa_circ_0035381
ASCRP001882	-3.6074826	3.6074826	-1.8509924	down	hsa_circRNA_101531	hsa_circ_0035445
ASCRP001967	-2.5725046	2.5725046	-1.3631737	down	hsa_circRNA_101618	hsa_circ_0000643
ASCRP002053	-3.1413549	3.1413549	-1.651387	down	hsa_circRNA_101706	hsa_circ_0005394
ASCRP002065	-2.0206257	2.0206257	-1.0148021	down	hsa_circRNA_101718	hsa_circ_0038082
ASCRP002102	-2.878952	2.878952	-1.5255437	down	hsa_circRNA_101756	hsa_circ_0038487
ASCRP002204	-2.1472822	2.1472822	-1.1025118	down	hsa_circRNA_101861	hsa_circ_0000714
ASCRP002223	-2.2011213	2.2011213	-1.1382387	down	hsa_circRNA_101881	hsa_circ_0040534
ASCRP002264	-2.2099414	2.2099414	-1.1440081	down	hsa_circRNA_101922	hsa_circ_0041103
ASCRP002286	-3.6412496	3.6412496	-1.8644336	down	hsa_circRNA_101945	hsa_circ_0041392
ASCRP002289	-2.5772576	2.5772576	-1.3658367	down	hsa_circRNA_101948	hsa_circ_0005479
ASCRP002293	-2.7677825	2.7677825	-1.4687306	down	hsa_circRNA_101952	hsa_circ_0041517
ASCRP002298	-2.8901699	2.8901699	-1.5311543	down	hsa_circRNA_101958	hsa_circ_0041555
ASCRP002309	-2.1649085	2.1649085	-1.114306	down	hsa_circRNA_101969	hsa_circ_0041821
ASCRP002325	-2.5383942	2.5383942	-1.3439161	down	hsa_circRNA_101988	hsa_circ_0042196
ASCRP002339	-3.1657599	3.1657599	-1.6625518	down	hsa_circRNA_102002	hsa_circ_0003505
ASCRP002380	-2.56205	2.56205	-1.3572986	down	hsa_circRNA_102045	hsa_circ_0002404
ASCRP002384	-126.88982	126.8898298	-6.9874326	down	hsa_circRNA_102049	hsa_circ_0043278

ASCRP002386	-71.5756	71.5755882	-6.1613957	down	hsa_circRNA_102051	hsa_circ_0006220
ASCRP002417	-2.5852751	2.5852751	-1.3703178	down	hsa_circRNA_102082	hsa_circ_0043947
ASCRP002451	-2.3480965	2.3480965	-1.2314917	down	hsa_circRNA_102119	hsa_circ_0044436
ASCRP002491	-2.9598503	2.9598503	-1.5655242	down	hsa_circRNA_102159	hsa_circ_0004006
ASCRP002553	-4.8312519	4.8312519	-2.2723971	down	hsa_circRNA_102225	hsa_circ_0046188
ASCRP002556	-2.2472194	2.2472194	-1.168141	down	hsa_circRNA_102228	hsa_circ_0000814
ASCRP002558	-2.2087279	2.2087279	-1.1432157	down	hsa_circRNA_102230	hsa_circ_0046215
ASCRP002565	-2.3430707	2.3430707	-1.2284005	down	hsa_circRNA_102237	hsa_circ_0046419
ASCRP002572	-2.0671147	2.0671147	-1.0476184	down	hsa_circRNA_102244	hsa_circ_0004063
ASCRP002643	-7.4705007	7.4705007	-2.9012049	down	hsa_circRNA_102324	hsa_circ_0047151
ASCRP002683	-2.3570486	2.3570486	-1.2369815	down	hsa_circRNA_102364	hsa_circ_0007006
ASCRP002717	-2.0624105	2.0624105	-1.0443315	down	hsa_circRNA_102398	hsa_circ_0004003
ASCRP002753	-2.1752594	2.1752594	-1.1211875	down	hsa_circRNA_102434	hsa_circ_0049138
ASCRP002761	-3.0566132	3.0566132	-1.611934	down	hsa_circRNA_102442	hsa_circ_0049271
ASCRP002764	-11.5913	11.5913991	-3.5349828	down	hsa_circRNA_102445	hsa_circ_0004552
ASCRP002765	-5.6459438	5.6459438	-2.4972148	down	hsa_circRNA_102446	hsa_circ_0049356
ASCRP002779	-2.3757429	2.3757429	-1.2483787	down	hsa_circRNA_102462	hsa_circ_0049700
ASCRP002787	-2.8407414	2.8407414	-1.5062675	down	hsa_circRNA_102470	hsa_circ_0049888
ASCRP002801	-2.3669244	2.3669244	-1.2430137	down	hsa_circRNA_102484	hsa_circ_0005571
ASCRP002802	-4.6505468	4.6505468	-2.2174003	down	hsa_circRNA_102485	hsa_circ_0050102
ASCRP002811	-2.1375547	2.1375547	-1.0959614	down	hsa_circRNA_102494	hsa_circ_0004066
ASCRP002823	-6.2286354	6.2286354	-2.6389161	down	hsa_circRNA_102509	hsa_circ_0006446
ASCRP002860	-3.3830066	3.3830066	-1.758306	down	hsa_circRNA_102547	hsa_circ_0008719
ASCRP002915	-7.097181	7.097181	-2.8272461	down	hsa_circRNA_102602	hsa_circ_0052318
ASCRP002928	-2.1719808	2.1719808	-1.1190113	down	hsa_circRNA_102615	hsa_circ_0008768
ASCRP002932	-38.672235	38.6722351	-5.2732262	down	hsa_circRNA_102619	hsa_circ_0000977
ASCRP002937	-5.1800207	5.1800207	-2.3729579	down	hsa_circRNA_102624	hsa_circ_0008261
ASCRP002944	-4.1780816	4.1780816	-2.0628407	down	hsa_circRNA_102631	hsa_circ_0005556
ASCRP003001	-3.8232086	3.8232086	-1.9347839	down	hsa_circRNA_102689	hsa_circ_0054021
ASCRP003033	-2.8577774	2.8577774	-1.5148935	down	hsa_circRNA_102721	hsa_circ_0054598
ASCRP003040	-2.2440999	2.2440999	-1.1661369	down	hsa_circRNA_102728	hsa_circ_0006110
ASCRP003053	-3.0565513	3.0565513	-1.6119048	down	hsa_circRNA_102741	hsa_circ_0003497

ASCRP003085	-2.0453255	2.0453255	-1.0323305	down	hsa_circRNA_102774	hsa_circ_0055412
ASCRP003092	-4.183422	4.183422	-2.0646835	down	hsa_circRNA_102782	hsa_circ_0055630
ASCRP003094	-2.5662816	2.5662816	-1.3596795	down	hsa_circRNA_102784	hsa_circ_0055734
ASCRP003115	-9.4367728	9.4367728	-3.2382936	down	hsa_circRNA_102806	hsa_circ_0008827
ASCRP003119	-2.85596	2.85596	-1.5139758	down	hsa_circRNA_102810	hsa_circ_0056285
ASCRP003171	-2.1177953	2.1177953	-1.0825631	down	hsa_circRNA_102863	hsa_circ_0057214
ASCRP003193	-3.1688948	3.1688948	-1.6639798	down	hsa_circRNA_102885	hsa_circ_0007422
ASCRP003256	-2.6898692	2.6898692	-1.427536	down	hsa_circRNA_102949	hsa_circ_0058792
ASCRP003257	-3.5309557	3.5309557	-1.8200587	down	hsa_circRNA_102950	hsa_circ_0058794
ASCRP003258	-2.109809	2.109809	-1.0771124	down	hsa_circRNA_102951	hsa_circ_0058805
ASCRP003285	-4.0304245	4.0304245	-2.0109318	down	hsa_circRNA_102978	hsa_circ_0004525
ASCRP003286	-13.127688	13.1276884	-3.714541	down	hsa_circRNA_102979	hsa_circ_0059151
ASCRP003297	-2.2957396	2.2957396	-1.198959	down	hsa_circRNA_102990	hsa_circ_0059369
ASCRP003298	-2.2922593	2.2922593	-1.1967702	down	hsa_circRNA_102991	hsa_circ_0006055
ASCRP003387	-2.319773	2.319773	-1.2139836	down	hsa_circRNA_103083	hsa_circ_0060848
ASCRP003412	-9.6135381	9.6135381	-3.2650675	down	hsa_circRNA_103108	hsa_circ_0061265
ASCRP003418	-2.3892707	2.3892707	-1.2565703	down	hsa_circRNA_103114	hsa_circ_0003323
ASCRP003428	-2.0638191	2.0638191	-1.0453165	down	hsa_circRNA_103124	hsa_circ_0001187
ASCRP003437	-2.6371091	2.6371091	-1.3989572	down	hsa_circRNA_103134	hsa_circ_0061749
ASCRP003440	-2.623799	2.623799	-1.3916572	down	hsa_circRNA_103137	hsa_circ_0061817
ASCRP003473	-2.2949932	2.2949932	-1.1984899	down	hsa_circRNA_103174	hsa_circ_0062522
ASCRP003492	-2.4468056	2.4468056	-1.2908995	down	hsa_circRNA_103194	hsa_circ_0062760
ASCRP003495	-2.5568317	2.5568317	-1.3543572	down	hsa_circRNA_103197	hsa_circ_0002954
ASCRP003509	-3.1835125	3.1835125	-1.6706194	down	hsa_circRNA_103211	hsa_circ_0063158
ASCRP003523	-7.1204458	7.1204458	-2.8319676	down	hsa_circRNA_103225	hsa_circ_0063331
ASCRP003568	-3.4233497	3.4233497	-1.7754087	down	hsa_circRNA_103270	hsa_circ_0064018
ASCRP003577	-2.9293041	2.9293041	-1.550558	down	hsa_circRNA_103279	hsa_circ_0008027
ASCRP003605	-19.58141	19.5814107	-4.2914128	down	hsa_circRNA_103307	hsa_circ_0064557
ASCRP003653	-2.1253267	2.1253267	-1.0876846	down	hsa_circRNA_103355	hsa_circ_0065243
ASCRP003659	-28.503021	28.5030211	-4.8330429	down	hsa_circRNA_103361	hsa_circ_0001296
ASCRP003682	-2.0974771	2.0974771	-1.0686551	down	hsa_circRNA_103384	hsa_circ_0065898
ASCRP003742	-6.5104812	6.5104812	-2.7027642	down	hsa_circRNA_103444	hsa_circ_0008797
ASCRP003754	-2.0194175	2.0194175	-1.0139392	down	hsa_circRNA_103456	hsa_circ_0067127

ASCRP003776	-2.2158663	2.2158663	-1.1478708	down	hsa_circRNA_103478	hsa_circ_0067492
ASCRP003839	-2.9987716	2.9987716	-1.5843716	down	hsa_circRNA_103541	hsa_circ_0068462
ASCRP003856	-2.1794604	2.1794604	-1.123971	down	hsa_circRNA_103558	hsa_circ_0068641
ASCRP003897	-2.0986733	2.0986733	-1.0694776	down	hsa_circRNA_103602	hsa_circ_0069152
ASCRP004014	-2.3177256	2.3177256	-1.2127098	down	hsa_circRNA_103723	hsa_circ_0070659
ASCRP004021	-7.193693	7.193693	-2.8467326	down	hsa_circRNA_103730	hsa_circ_0005654
ASCRP004040	-2.5615802	2.5615802	-1.357034	down	hsa_circRNA_103749	hsa_circ_0005480
ASCRP004074	-2.1704455	2.1704455	-1.1179912	down	hsa_circRNA_103784	hsa_circ_0008903
ASCRP004091	-12.389073	12.3890739	-3.6309964	down	hsa_circRNA_103801	hsa_circ_0071989
ASCRP004108	-9.4247661	9.4247661	-3.2364568	down	hsa_circRNA_103818	hsa_circ_0072279
ASCRP004170	-3.1998767	3.1998767	-1.6780163	down	hsa_circRNA_103890	hsa_circ_0007158
ASCRP004181	-2.0407304	2.0407304	-1.0290856	down	hsa_circRNA_103901	hsa_circ_0004539
ASCRP004196	-2.3089114	2.3089114	-1.2072128	down	hsa_circRNA_103916	hsa_circ_0005540
ASCRP004224	-6.7481584	6.7481584	-2.7544938	down	hsa_circRNA_103944	hsa_circ_0005105
ASCRP004292	-3.1344699	3.1344699	-1.6482215	down	hsa_circRNA_104017	hsa_circ_0004004
ASCRP004315	-10.876500	10.8765004	-3.4431425	down	hsa_circRNA_104040	hsa_circ_0075410
ASCRP004318	-25.866360	25.8663605	-4.6930052	down	hsa_circRNA_104044	hsa_circ_0075447
ASCRP004324	-2.0248507	2.0248507	-1.0178155	down	hsa_circRNA_104050	hsa_circ_0075504
ASCRP004365	-2.1297862	2.1297862	-1.0907086	down	hsa_circRNA_104092	hsa_circ_0076041
ASCRP004372	-4.7196459	4.7196459	-2.2386786	down	hsa_circRNA_104099	hsa_circ_0076092
ASCRP004391	-2.3458404	2.3458404	-1.2301049	down	hsa_circRNA_104118	hsa_circ_0003563
ASCRP004394	-2.3542025	2.3542025	-1.2352384	down	hsa_circRNA_104121	hsa_circ_0076767
ASCRP004399	-2.6429312	2.6429312	-1.4021389	down	hsa_circRNA_104126	hsa_circ_0076798
ASCRP004408	-5.6804578	5.6804578	-2.5060072	down	hsa_circRNA_104135	hsa_circ_0007874
ASCRP004421	-4.189736	4.189736	-2.0668593	down	hsa_circRNA_104148	hsa_circ_0008236
ASCRP004434	-2.2365311	2.2365311	-1.1612628	down	hsa_circRNA_104161	hsa_circ_0077514
ASCRP004466	-4.5285163	4.5285163	-2.1790385	down	hsa_circRNA_104193	hsa_circ_0077930
ASCRP004467	-3.4985892	3.4985892	-1.8067733	down	hsa_circRNA_104194	hsa_circ_0004712
ASCRP004495	-15.195752	15.1957526	-3.9255962	down	hsa_circRNA_104223	hsa_circ_0078277
ASCRP004499	-4.1100532	4.1100532	-2.0391571	down	hsa_circRNA_104227	hsa_circ_0078297
ASCRP004529	-4.7329956	4.7329956	-2.2427536	down	hsa_circRNA_104258	hsa_circ_0078605
ASCRP004531	-2.1026613	2.1026613	-1.0722165	down	hsa_circRNA_104260	hsa_circ_0078610
ASCRP004540	-9.8493043	9.8493043	-3.3000218	down	hsa_circRNA_104270	hsa_circ_0001666

ASCRP004553	-2.0212562	2.0212562	-1.0152522	down	hsa_circRNA_104288	hsa_circ_0079163
ASCRP004574	-2.4918191	2.4918191	-1.3171993	down	hsa_circRNA_104310	hsa_circ_0079385
ASCRP004578	-2.5540806	2.5540806	-1.352804	down	hsa_circRNA_104315	hsa_circ_0079480
ASCRP004605	-5.3411322	5.3411322	-2.4171456	down	hsa_circRNA_104342	hsa_circ_0003162
ASCRP004618	-2.7682052	2.7682052	-1.4689509	down	hsa_circRNA_104356	hsa_circ_0079996
ASCRP004660	-5.953838	5.953838	-2.57382	down	hsa_circRNA_104400	hsa_circ_0006944
ASCRP004661	-8.1671944	8.1671944	-3.0298406	down	hsa_circRNA_104401	hsa_circ_0005513
ASCRP004672	-2.038592	2.038592	-1.0275731	down	hsa_circRNA_104416	hsa_circ_0080822
ASCRP004728	-2.6710991	2.6710991	-1.4174335	down	hsa_circRNA_104474	hsa_circ_0082179
ASCRP004749	-7.1880188	7.1880188	-2.8455942	down	hsa_circRNA_104495	hsa_circ_0001973
ASCRP004822	-2.6847024	2.6847024	-1.4247622	down	hsa_circRNA_104569	hsa_circ_0083619
ASCRP004825	-3.5464109	3.5464109	-1.8263597	down	hsa_circRNA_104572	hsa_circ_0007618
ASCRP004837	-2.4811653	2.4811653	-1.3110179	down	hsa_circRNA_104584	hsa_circ_0083861
ASCRP004848	-9.729683	9.729683	-3.2823928	down	hsa_circRNA_104595	hsa_circ_0008016
ASCRP004905	-2.431503	2.431503	-1.2818483	down	hsa_circRNA_104652	hsa_circ_0084984
ASCRP004919	-2.8524606	2.8524606	-1.512207	down	hsa_circRNA_104666	hsa_circ_0008496
ASCRP004929	-5.0927009	5.0927009	-2.348431	down	hsa_circRNA_104676	hsa_circ_0008035
ASCRP004946	-4.8838623	4.8838623	-2.2880225	down	hsa_circRNA_104694	hsa_circ_0007934
ASCRP004955	-4.7353436	4.7353436	-2.2434691	down	hsa_circRNA_104703	hsa_circ_0008305
ASCRP004962	-4.0493803	4.0493803	-2.0177011	down	hsa_circRNA_104710	hsa_circ_0006421
ASCRP005005	-5.4734861	5.4734861	-2.45246	down	hsa_circRNA_104755	hsa_circ_0086686
ASCRP005009	-2.7832005	2.7832005	-1.4767448	down	hsa_circRNA_104759	hsa_circ_0007367
ASCRP005052	-2.1981449	2.1981449	-1.1362865	down	hsa_circRNA_104803	hsa_circ_0087354
ASCRP005077	-2.1231917	2.1231917	-1.0862346	down	hsa_circRNA_104829	hsa_circ_0087565
ASCRP005162	-2.4599919	2.4599919	-1.2986535	down	hsa_circRNA_104915	hsa_circ_0088485
ASCRP005176	-2.3381937	2.3381937	-1.2253945	down	hsa_circRNA_104929	hsa_circ_0088807
ASCRP005177	-2.9440804	2.9440804	-1.5578171	down	hsa_circRNA_104930	hsa_circ_0002675
ASCRP005181	-5.2670897	5.2670897	-2.397006	down	hsa_circRNA_104934	hsa_circ_0007311
ASCRP005233	-2.2268475	2.2268475	-1.1550027	down	hsa_circRNA_104986	hsa_circ_0090080
ASCRP005260	-3.6411897	3.6411897	-1.8644099	down	hsa_circRNA_105013	hsa_circ_0001936
ASCRP005301	-2.1716185	2.1716185	-1.1187707	down	hsa_circRNA_400005	hsa_circ_0092329
ASCRP005305	-2.2572514	2.2572514	-1.1745671	down	hsa_circRNA_400009	hsa_circ_0092340
ASCRP005308	-2.2559061	2.2559061	-1.173707	down	hsa_circRNA_400012	hsa_circ_0092339

ASCRP005315	-2.4516068	2.4516068	-1.2937276	down	hsa_circRNA_400019	hsa_circ_0092342
ASCRP005316	-3.3445007	3.3445007	-1.7417909	down	hsa_circRNA_400020	hsa_circ_0092372
ASCRP005317	-2.382044	2.382044	-1.2522001	down	hsa_circRNA_400021	hsa_circ_0092306
ASCRP005323	-3.1445995	3.1445995	-1.6528763	down	hsa_circRNA_400027	hsa_circ_0092367
ASCRP005329	-13.43710	13.4371059	-3.7481505	down	hsa_circRNA_400033	hsa_circ_0092360
ASCRP005386	-2.8828515	2.8828515	-1.5274965	down	hsa_circRNA_400093	hsa_circ_0092290
ASCRP005388	-6.3102236	6.3102236	-2.6576911	down	hsa_circRNA_400095	hsa_circ_0092321

Supplemental Table 2. circNOL10-sponged miRNA

miRNA			
hsa-miR-1	hsa-miR-4259	hsa-miR-29b	hsa-miR-559
hsa-miR-103	hsa-miR-4263	hsa-miR-29c	hsa-miR-571
hsa-miR-107	hsa-miR-452	hsa-miR-3132	hsa-miR-584
hsa-miR-1208	hsa-miR-495	hsa-miR-330-3p	hsa-miR-613
hsa-miR-124	hsa-miR-506	hsa-miR-338-3p	hsa-miR-615-5p
hsa-miR-146b-3p	hsa-miR-513a-3p	hsa-miR-3664	hsa-miR-634
hsa-miR-149	hsa-miR-524-3p	hsa-miR-3673	hsa-miR-760
hsa-miR-1972	hsa-miR-525-3p	hsa-miR-3674	hsa-miR-767-5p
hsa-miR-206	hsa-miR-548g	hsa-miR-380	hsa-miR-877

Supplemental Table 3. circNOL10-sponged miRNA pathways

Pathway	p value	Gene number	p value (-log10)	Gene rich
Focal adhesion	2.70463E-38	105	37.56789198	0.527638191
Wnt signaling pathway	2.12349E-33	84	32.67294937	0.575342466
Ubiquitin mediated proteolysis	3.28338E-27	71	26.48367898	0.518248175
Insulin signaling pathway	7.90678E-26	70	25.10200013	0.510948905
Adherens junction	2.42592E-21	43	20.61512353	0.597222222
Mrna surveillance-pathway	1.89152E-20	51	19.72318906	0.56043956
Mapk signaling pathway	4.2491E-20	122	19.37170315	0.413559322
Erbb signaling pathway	5.04E-19	47	18.29756946	0.552941176
Prostate-cancer	1.1E-18	49	17.95860731	0.505154639

Pathways in cancer	7.23633E-18	158	17.1404814	0.300380228
Transcriptional misregulation in cancer	4.0355E-17	91	16.39410254	0.489247312
Neurotrophin signaling pathway	6.26088E-17	60	16.20336462	0.504201681
Pancreatic cancer	1.58145E-16	40	15.80094453	0.533333333
Regulation of actin cytoskeleton	3.20326E-16	100	15.49440808	0.469483568
Adipocytokine-signaling pathway	1.85536E-15	38	14.73157228	0.550724638
B cell receptor signaling pathway	4.24831E-15	39	14.37178431	0.549295775
Melanoma	6.27556E-15	38	14.20234786	0.527777778
RNA degradation	8.87206E-15	38	14.05197548	0.481012658
Colorectal cancer	8.87206E-15	36	14.05197548	0.418604651
Small cell lung cancer	1.2231E-14	42	13.91253945	0.451612903
Dopaminergic synapse	1.65462E-14	64	13.78130068	0.488549618
RNA transport	4.17259E-14	72	13.37959481	0.421052632
mTOR signaling pathway	6.84041E-14	36	13.16491812	0.238410596
Axon guidance	7.40458E-14	64	13.13049939	0.365714286
Protein processing in endoplasmic reticulum	1.34E-12	79	11.8728952	0.478787879
Endometrial cancer	2.12E-12	31	11.67366414	0.534482759
Gap junction	5.82E-11	43	10.23507702	0.488636364
Hedgehog signaling pathway	1.62E-10	27	9.790484985	0.574468085
Thyroid cancer	1.41E-09	21	8.850780887	0.567567568
Chronic myeloid leukemia	2.37E-09	36	8.625251654	0.473684211
Acute-myeloid leukemia	2.71E-09	29	8.567030709	0.439393939
Prion diseases	5.77E-09	16	8.238824187	0.457142857
Gnrh signaling pathway	8.05E-09	42	8.09420412	0.451612903

Detailed Experimental Section

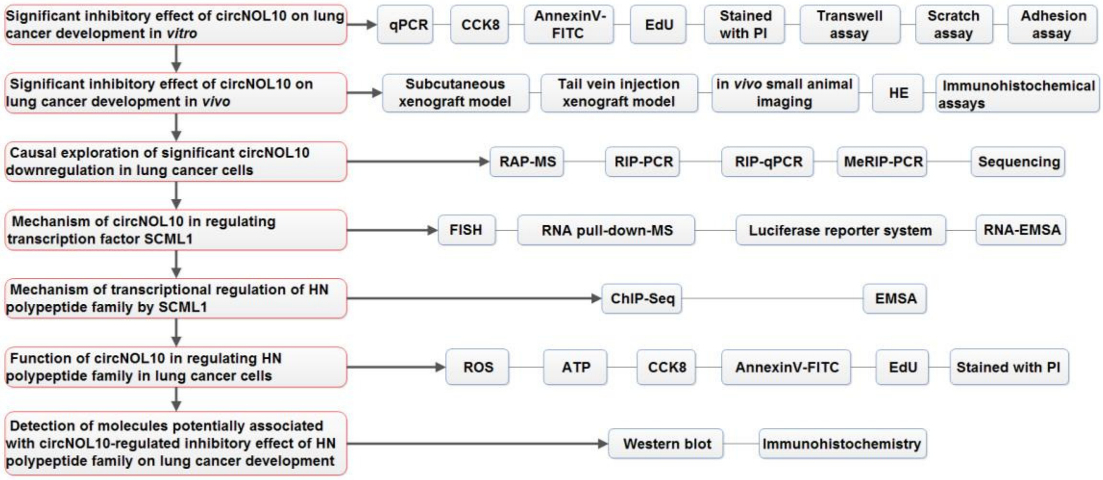

Supplemental Figure 7. Schematic figure of the detailed experimental methods and flow chart.

RNA Extraction, Reverse Transcription, and qPCR: Total RNA was extracted from cells and tissues using TRIzol reagent, according to the manufacturer's instructions (Ambion/Invitrogen Co., Carlsbad, CA, USA). The detailed procedures were as follows. 1×106cells were lysised with 1ml TRIzol reagent，add 200μl of chloroform per 1mL samples, followed by incubation for 10 min and centrifugation for 15 min at 12,000 ×g at 4°C. The aqueous phase containing the RNA was transferred to a new tube and 0.5 ml of isopropanol was added, followed by incubation for 10 min and centrifugation for 10 min at 12,000 ×g at 4 °C. The pellet was resuspended in 1 ml of 75% ethanol. The sample was then briefly vortexed, centrifuged for 5 min at 7500 ×g at 4°C, and resuspended in 1 ml of absolute ethyl alcohol. The sample was briefly vortexed again and centrifuged for 7 min at 7500 ×g at 4°C. The RNA pellet was then air-dried for 10 min, resuspended in 20 µl of RNase-free water, and incubated in a heat block at 58°C for 10 min. The RNA concentration was then measured and the quality and concentration of the extracted RNAs were examined using a NanoDrop 1000 Spectrophotometer (NanoDrop Technologies, Wilmington, DE, USA). For quantification of circRNAs, RNase R (EpicentreBio, Madison, WI, USA) was added to digest linear RNAs during RNA extraction, using 3U RNase R enzyme per 1000 ng RNA and incubation for 10 min at 37℃. RNAs (circRNA, mRNA, and pre-mRNA) were reverse transcribed into cDNAs using a Goscript™ Reverse Transcription System (Promega, Madison, WI, USA) as follows. Nuclease-free water was added to

1000 ng RNA and 1 µl random primer to a volume of 10 µl and mixed well. The mixture was then thermally denatured for 5 min at 70°C. Nuclease-free water 1.5 µl, 5× Reaction Buffer 4 µl, $MgCl_2$ 2 µl, PCR Nucleotide Mix 1 µl, reverse transcriptase 1 µl, and ribonuclease inhibitor 0.5 µl were added to a final volume of 20 µl and mixed well to anneal for 5 min at 25°C, followed by extension for 60 min at 42°C, inactivation for 15 min at 70°C, and chilling at 4°C. qPCR was carried out using Go Taq® qPCR Master Mix (Promega). GAPDH was used as an internal reference gene. Relative expression levels of the genes were determined using the $2^{-\Delta\Delta Ct}$ method, as follows. Nuclease-free water 7 µl, forward primer 0.4 µl, reverse primer 0.4 µl, GoTaq qPCR Master Mix 10 µl, CXR 0.2 µl, and cDNA 2 µl were mixed well, Select SYBR as the detection dye for the entire plate. Hot-start activation was carried out for 2 min at 95°C for one cycle, followed by denaturation for 15 s at 95°C and extension for 60 s at 60°C for 40 cycles.

Cell Culture and Human Tissue Sample Collection: The cells used in this study were all cultured in strict accordance with the cell culture conditions and methods recommended by the American Type Culture Collection (https://www.atcc.org). BEAS-2B cells were cultured in serum-free BEGM (Lonza/Clonetics Co, Kit Catalog No. CC-3170, Walkersville, MD, USA). H460 cells were cultured in RPMI-1640 (Hyclone, Logan, Australia) and A549 cells in F12K (ThermoFisher Scientific, Carlsbad, CA, USA), both supplemented with 10% fetal bovine serum (Every Green, Hangzhou, China). The cells were cultured at 37°C and 5% CO_2 under saturated humidity. Human tissue samples were provided by the tissue bank of the Institute of Chemical Carcinogenesis of Guangzhou Medical University and were derived from individuals recruited from Guangzhou City. Informed consent was obtained from all patients before sample collection. This study was approved by the Medical Ethics Committee of Guangzhou Medical University.

Expression Vectors: The circNOL10 overexpression vector used for transient transfection was pcircRNA1.1 (BersinBio, Guangzhou, China) and the vector used for stable transfection was pLV-circRNA1.1 (BersinBio). The eukaryotic protein expression vector was pcDNA3.1 (Invitrogen, ThermoFisher Scientific, Carlsbad, CA, USA) and the prokaryotic protein expression vector was pGEX-6P-1 (GE Healthcare,

Boston, MA, USA). The luciferase reporter vector was psiCHECK™-2 Vector (Promega).

Gene Silencing and Overexpression: Transient siRNA transfection was used for in vitro functional studies. siRNAs targeting circRNA and mRNA were designed and synthesized and their specificities tested. In this study, three siRNAs were designed for each gene, and the two with the higher silencing efficiencies were chosen for subsequent experiments. The transfection reagent used in the silencing experiments was Ribo FECT™ CP Transfection Kit (166T) (RiboBio, Guangzhou, China). Overexpression vectors were constructed for targeting genes (circRNA and mRNA). Stable transfection was used for in vivo studies. A specific lentiviral vector was constructed and packaged. The transfection reagent used in the overexpression experiments was EndoFectin-Lenti (GeneCopoeia, Rockville, MD, USA). All the experiments were conducted in strict accordance with the relevant manufacturer's instructions. The eukaryotic protein expression vector used in this study was pcDNA3.1 (Invitrogen), the prokaryotic protein expression vector was pGEX-6P-1 (GE Healthcare, Boston, MA, USA), and the luciferase reporter vector was psiCHECK™-2 Vector (Promega).

Cell Viability Detected using the CCK8 Assay: Cell viability was detected using a Cell Counting Kit-8 (Beyotime Biotechnology, Shanghai, China). Cells in logarithmic phase were collected and seeded into 96-well plates, with 2000 cells/100 μl well. The cells were cultured in 5% CO_2 at 37°C overnight followed by gene silencing or overexpression treatment. The medium was replaced with fresh medium 6 h after transfection, and 10 μl of CCK8 solution was added to each well after 48 h of transfection, followed by incubation for a further 1–4 h. The absorbance of each well was measured at 450 nm using a microplate reader (BioTex, Houston, TX, USA). The reaction was terminated according to the absorbance value.

Apoptosis Detected with Flow Cytometry (FCM): Apoptosis was detected using an Annexin V-fluorescein isothiocyanate (FITC) apoptosis detection kit (KeyGen Biotech, Nanjing, China). A total of 5×10^5 cells were collected from each sample 48 h after transfection and resuspended in 100 μl of 1× Binding Buffer, followed by the

addition of 5 μl Annexin V-FITC and 5 μl propidium iodide staining solution and mixing. The reaction was carried out for 10 min in the dark at room temperature, and 400 μl 1×Binding Buffer was then added and mixed evenly. The cells were detected within 1 h using a Guava® easyCyte flow cytometry system (Millipore, Billerica, MA, USA).

Cell Proliferation Detected using the EdU Assay: The EdU assay was carried out using a Cell-Light™ EdU Apollo® 567 In Vitro Imaging Kit (RiboBio, Guangzhou, China). Cells were seeded into 12-well plates, and EdU solution (Reagent A) was diluted with cell culture medium at a ratio of 2500:1, and then added into the plate. The cells were cultured at 37°C in 5% CO_2 for 24 h, the medium was then discarded, and the cells were washed twice with phosphate-buffered saline (PBS) for 5 min each and then fixed with 4% paraformaldehyde for 30 min at room temperature. The fix solution was then discarded and the cells were washed with 2 mg/ml glycine solution for 5 min followed by PBS for 5 min, and then washed with 0.5% TritonX-100/PBS for 10 min followed by PBS for 5 min. The 1× Apollo staining reaction solution was prepared, added into the wells, and incubated for 30 min in the dark at room temperature. The staining solution was then removed and the cells were washed twice in 0.5 TritonX-100/PBS for 10 min each, followed by methanol for 5 min and PBS for 5 min. The 1x Hoechst33342 reaction solution was prepared by diluting the kit F reagent with deionized water at a ratio of 100:1, adding it into the wells, and incubating for 30 min in the dark at room temperature. The reaction solution was then removed and the cells were washed three times with PBS for 5 min each and then observed and imaged using an inverted fluorescence microscope (DMI3000 B, Leica, Wetzlar, Germany).

Cell Cycle Detected with FCM: Cell cycle analysis was performed using a Cell Cycle Detection kit (KeyGen Biotech). At 48 h after transfection, 5×10^5 cells were collected from each sample, added into 2 ml centrifuge tubes, and washed three times with 1× precooled PBS. After centrifugation, the cell pellets were collected and the supernatants were discarded. The cells were resuspended in 0.1 ml 1× PBS and rapidly added into 1.9 ml 75% precooled ethanol and mixed well by pipetting. The cells were then stored at −20°C overnight. The ethanol-fixed cells were centrifuged

and the supernatants were discarded. The cells were washed three times with 1× PBS to remove residual ethanol, resuspended in 0.1 ml solution containing 0.1 mg/ml RNase A, and incubated at 37°C in a water bath for 15 min, followed by the addition of 0.4 ml propidium iodide and incubation at room temperature for 10 min. Cell cycle was detected using a Guava® easyCyte flow cytometry system (Millipore).

Cell Invasion Detected using the Transwell Migration Assay: The transwell chambers (BD, Corning, NY, USA) were coated with extracellular matrix and hydrated in serum-free medium overnight. Transfected cells were digested with trypsin to prepare a cell suspension, counted, and adjusted to 1.2×10^4/ml. Medium containing 15% fetal bovine serum (700 µl) was added into the lower chamber and the upper chamber was placed gently on top of the lower chamber, avoiding air bubbles, then 200 µl of cell suspension was added into the upper chamber and incubated for 24 h. After incubation, the upper chamber was removed and placed in a clean 24-well plate and washed three times with PBS three times, followed by the addition of 200 µl 100% methanol for 15 min to fix the cells. The methanol was discarded and 200 µl of crystal violet was added for 15 min to stain the cells. The crystal violet was then discarded and the cells were washed twice with PBS. Unmigrated cells were removed using a cotton swab and migrated cells were observed and photographed under a light microscope.

Cell Migration Detected using the Scratch Assay: Reference lines were drawn across the wells on the outer bottom of a six-well plate using a marker pen. After 48 h of transfection, 1×10^6 cells were then seeded into each well and incubated overnight. Scratches were made perpendicular to the reference lines using a pipette tip. The cells were washed three times with PBS to remove loose cells, followed by the addition of serum-free medium and incubation at 37°C in 5% CO_2 for 24 h. The cells were then observed and photographed under a light microscope.

Cell Adhesion Test: Plates were pre-plated with 10 µg/ml fibronectin (FN) (BD, Corning) 70 µl/well at 4°C overnight, washed three times with PBS, and then blocked with 1% BSA (Solarbio, Beijing, China) at 37°C for 1 h. The transfected cells were digested and suspended in serum-free medium. The cell concentration was adjusted to

5×10^5/ml and the cells were seeded into the FN pre-spread 96-well plates. Each well contained ~5000 cells and three replicate wells were set up for each group. Cells were incubated for 1 h at 37°C, and non-adherent cells were then washed off with PBS. CCK8 solution 10 μl was added into each well and incubated for 1–4 h, after which the cell culture was terminated and the viability of the adherent cells was tested to reflect their adhesion ability.

Construction of Lung Cancer Xenograft Models in Nude Mice: We constructed a subcutaneous xenograft model and a tail vein injection xenograft model in nude mice. This study was approved by the Animal Experiment Ethics Committee of Guangzhou Medical University. All mice were kept in a specific-pathogen-free animal room in the Experimental Animal Center of Guangzhou Medical University. To create the nude mouse subcutaneous xenograft model, female BALB/c-nu/nu mice were purchased from Guangdong Medical Laboratory Animal Center. H460 cells (circNOL10 stably overexpressed, green fluorescent protein-labeled) were washed with PBS and adjusted to a final cell concentration of 5×10^6/0.1 ml. The mice were then inoculated subcutaneously with 0.1 ml of cell solution into the right axillary region. Pressure was applied to the injection site using an alcohol-soaked cotton ball for a few seconds. All operations were finished within 30 min after harvesting cells and strictly followed aseptic principles. For the tail vein injection xenograft model, mice and cell types were obtained as mentioned above. Cells were adjusted to 1×10^7/0.1 ml and 0.1 ml was injected into the tail vein of the model mice. After cell injection, bioluminescence imaging was performed using an in vivo small animal imaging system (Night OWL II LB983 NC100, Berthold Technologies, Stuttgart, Germany) at 1, 3, 5, 7, 14, and 21 days. After imaging, all mice continued to be fed until 21 days after inoculation and were then killed humanely.

Immunohistochemistry: Tissue samples were fixed, dehydrated through graded ethanols, paraffin-embedded, sectioned, dried, and baked. For immunohistochemical staining, sections were treated sequentially with xylenes I–III for 15 min each, followed by, absolute ethanols I–II for 5 min each, 85% alcohol and 75% alcohol for 5 min each, and then washed with distilled water. The tissue sections were placed in a repair box filled with citrate antigen repair buffer (pH 6.0) followed by heating in a

pressure cooker with a certain amount of water. Heating was stopped when water vapor began to escape, and the pressure was then released. The sections were then placed in the repair box, being careful to avoid excessive evaporation of the buffer. The sections were allowed to cool naturally, and washed three times with PBS on a decolorizing shaker, for 5 min each. The sections were then placed in 3% H_2O_2 solution, incubated at room temperature for 25 min in a dark environment, washed three times with PBS for 5 min each, covered evenly with 3% BSA, and then blocked at room temperature for 30 min. The blocking solution was removed gently and primary antibody solution in PBS was dropped onto the sections and incubated in a humid chamber at 4°C overnight. The sections were then washed three times with PBS on a decolorizing shaker for 5 min each, followed by incubation with horseradish peroxidase-labeled secondary antibody solution (from the same species as the primary antibody) at room temperature for 50 min followed by three washes with PBS for 5 min each. Freshly-prepared diaminobenzidine (DAB) staining solution was then dropped onto the samples and the staining time was controlled by observing the sections under a microscope. Positive staining was indicated by brown-yellow color. The sections were then washed in running distilled water to terminate the reaction, counter-stained with hematoxylin for 3 min, and washed again with running water. The sections were immersed in 1% hydrochloric acid alcohol for several seconds and then washed again with running water. Ammonia water was used to blue the hematoxylin. Sections were then dehydrated by sequential treatment with 75% alcohol, 85% alcohol, absolute ethanol I, absolute ethanol, and xylene for 5 min each. The sections were dried and mounted with neutral gum and observed and photographed under a light microscope.

Hematoxylin–Eosin Staining: Sample preparation and sectioning were performed as above. The sections were treated sequentially with xylene I and xylene II for 20 min each, absolute ethanol I and absolute ethanol II for 10 min each, and 95%, 90%, 80%, and 70% alcohol for 5 min each, and then washed with distilled water. The sections were immersed in hematoxylin for 5 min, washed with running water, and then treated with 1% hydrochloric acid alcohol for several seconds, followed by washing with running water, bluing in aqueous 0.6% ammonia solution, and washing with running water. The sections were then stained with eosin solution for 1 min and

dehydrated by sequential treatment with 95% alcohol I, 95% alcohol II, absolute ethanol I, absolute ethanol II, and xylene for 5 min each. The sections were then dried and mounted with neutral gel, and observed and photographed under a light microscope.

RNA Antisense Purification (RAP) and Mass Spectrometry (MS): RAP was performed using a RAP kit (BersinBio, Guangzhou, China) according to the manufacturer's instructions. The main steps of the experiment included cross-linking cells, collecting cell pellets, homogenization, removal of the DNA, preparation of the probe, preparation of the beads, hybridization and capture, protein elution, protein precipitation, protein dissolution, and quality control analysis. The obtained proteins were then used for western blot or MS. MS was performed in accordance with standard procedures using a Q Exactive mass spectrometer (ThermoFisher Scientific). The proteins were first digested to produce peptide fragments, and the fragments were then dissolved, vortexed thoroughly, and centrifuged at 16,363 g at 4°C for 10 min. The supernatants were transferred to a loading tube for MS identification.

Western Blot: Proteins were extracted using a total protein extraction kit (KeyGen Biotech), according to the manufacturer's instructions, and the extracted proteins were quantified. For electrophoresis, the concentrations of the loaded protein samples were adjusted to the same level. The loading samples were prepared by mixing with ×5 loading buffer (β-mercaptoethanol was added) at a ratio of 4:1 and the proteins were denatured at 100°C for 5 min and then stored at −80°C. The proteins were then loaded onto a discontinuous sodium dodecyl sulfate-polyacrylamide gel at 40–60 μg/well and subjected to electrophoresis at 100 V, followed by 120 V when the samples reached the separation gel. A marker with a molecular weight of 16–220 kD was included in the gel. The proteins were transferred to a polyvinylidene difluoride membrane (Millipore) using a wet-transfer device (Bio-Rad, CA, USA) under 200 mA constant current flow, for a time adjusted according to the molecular weight of the target proteins. After transfer, the membrane was rinsed with TBST for 1–2 min and blocked with 5% skimmed milk for 60 min. The membrane was then cut appropriately as determined by the marker. Primary antibody was diluted according to the instructions, and the membrane was placed in the primary antibody solution at 4°C overnight,

followed by three washes with TBST for 15 min each. Secondary antibody was also diluted according to the kit instructions and incubated with the membrane for 1 h, followed by three washes with TBST for 15 min each. Finally, the membrane was stained using BeyoECL Plus chemiluminescence reagents.

RNA Immunoprecipitation (RIP): Cells were lysed and sonicated, followed by removal of DNA and balance protein A/G, and divided into three groups of 0.8 ml each: Input samples were stored at −20°C until needed; immunoprecipitation (IP) and IgG samples were added to the test antibodies or same amount of IgG (negative control antibody) and incubated in a vertical mixer at 4°C for 16 h overnight, followed by the addition of 20 µl of protein A/G beads, and incubation in a vertical mixer at 4°C for 1 h. The magnetic beads were collected and the supernatants were discarded, and 0.5 ml of polysome washing buffer 1 and 5 µl of dithiothreitol (DTT) were added to the IP and IgG samples, respectively, followed by washing three times. The magnetic beads were then collected and the supernatant was removed, and 94.5 µl of polysome washing buffer 1, 0.5 µl of DNase salt stock, and 5 µl of DNase (5 U) were added to the IP and IgG samples, respectively, and incubated for 5 min at 37°C. The magnetic beads were then collected and the supernatant was removed, and the IP and IgG samples were washed with 0.5 ml of polysome washing buffer 2 and 5 µl DTT, and incubated for 5 min at 4°C. The magnetic beads were collected and the supernatant was removed, and 100 µl polysome elution buffer was added, and the beads were then resuspended in 1 µl DTT and 1 µl proteinase K. One microliter of DTT and 2 µl proteinase K were added to the input samples and incubated at 55°C for 1 h. The RNAs were eluted and the magnetic beads were collected, and the supernatant was transferred to a clean RNase-free centrifuge tube. RNAs were extracted and subjected to reverse transcription, PCR, and qPCR experiments.

Methylated RNA Immunoprecipitation (MeRIP): MeRIP was performed using a MeRIP kit (BersinBio). First, total RNAs were extracted and 10 µl RNA samples were used as the input samples, and stored at −80°C until use. For IP, m6A-specific antibody, 200 µl 1×IP buffer and 2 µl RNase inhibitor was added to the remaining RNA samples and incubated in a vertical mixer at 4°C for 2–4 h. The protein A/G beads were prepared by washing 30 µl beads for each MeRIP sample twice with 1 ml

1× IP buffer, followed by 100 μl 1× IP buffer (containing 0.5 mg/ml BSA) to block the protein A/G beads at 4°C for 2 h. The blocked beads were then washed twice with 100 μl 1× IP buffer. Protein A/G binding to the antibody was performed by incubating the prepared protein A/G beads with the sample–antibody hybrid solution in a vertical mixer at 4°C for 2–4 h, washing three times with 500 μl 1× IP buffer, and incubation with 200 μl 1× IP buffer (plus 1 μl proteinase K) at 50°C for 45 min, with mixing every 10 min. RNAs were extracted and subjected to reverse transcription, PCR, and qPCR experiments.

RNA-Fluorescence in Situ Hybridization (FISH): The cells were seeded on coverslips in 12-well plates and cultured for 24 h to achieve 70% confluence. The medium was discarded, and the cells were washed twice with PBS for 5 min each. The cells were then fixed with 4% paraformaldehyde at room temperature for 30 min, the fixing solution was discarded, and the cells were washed twice with PBS for 5 min each. The cells were digested with 20 μg/ml proteinase K (Sangon Biotech, Shanghai, China) at 37°C for 5 min, washed briefly with PBS, fixed with 1% paraformaldehyde at room temperature for 10 min, and then washed twice with PBS for 5 min each. The cell coverslips were then washed sequentially with 70%, 85%, and 100% ethanol for 3 min each. The prepared 6-FAM-labeled fluorescent probes (Sangon Biotech) were dropped onto the coverslips and denatured for 3 min at 73°C, followed by transferring the coverslips to the dark at 37°C and incubating for 16 h. The next day, the cells were washed with washing solution 1 (50% formamide/2× SSC) at 43°C for 5 min, followed by washing solution 2 (0.1 Triton X-100/2× SSC) at 37°C for 5 min, and finally, 2× SSC at room temperature for 5 min. Nuclei were counterstained with 4',6-diamidino-2-phenylindole (DAPI) and the cells were washed with PBS. The coverslips were removed from the 12-well plates, air-dried, and mounted with anti-fluorescence attenuation agent. The cells were then observed and photographed using a laser confocal microscope.

RNA Pull-down: RNA pull-down experiments were carried out using an RNA pull-down kit (BersinBio). Full-length and truncated biotinylated RNA pull-down probes were designed and synthesized, and the experiments were conducted in strict accordance with the instructions of the kit manufacturer. The main steps included

formation of RNA secondary structure, preparation of probe–magnetic bead complex, extraction of total proteins, removal of nucleic acids from protein samples and pre-washing, acquisition of RNA-binding proteins by RNA pull-down, collection of RNA-binding proteins, protein denaturation, and analysis of the obtained proteins. The acquisition and collection of RNA-binding proteins were core steps. Protein acquisition by RNA pull-down was achieved as follows: cell extracts were mixed with the probe–magnetic bead complex followed by the addition of 0.5 ml RIP buffer, 5 µl protease inhibitor cocktail, 5 µl DTT, 5 µl RNase inhibitor, and 5 µl poly(dI.dC), and then allowed to bind with gentle rotation at 25°C for 2 h After standing for 1 min, the magnetic beads were collected and the supernatants were discarded, and 1 ml pre-cooled NT2 buffer was added and mixed fully at 4°C for 5 min. The samples were then placed on a magnetic stand for 1 min to collect the magnetic beads, the supernatant was discarded, and the magnetic beads were washed four times. RNA-binding proteins were collected as follows: 30 µl of urea CHAPS buffer and 0.3 µl of DTT were added to the magnetic bead solution in the RNA-pull-down and negative control groups, and then eluted by incubation at 37°C for 2 h. The magnetic beads were collected as described above and the supernatants were transferred to clean centrifuge tubes. The elution was repeated several times and the obtained proteins were stored at −80°C for future use.

Immunocytochemistry: The cells were seeded on coverslips in 12-well plates and cultured for 24 h to achieve 70% confluence. The culture medium was discarded and the cells were washed twice with PBS for 5 min each, and then fixed with 4% paraformaldehyde for 30 min at room temperature. The fixation solution was discarded and the cells were washed with PBS. The coverslips were immersed in 0.6% H_2O_2/methanol mixture at room temperature for 30 min to inactivate endogenous peroxidase, and then washed twice with distilled water for 5 min each. Blocking solution (5% BSA) was dropped onto the samples at room temperature for 20 min and then discarded. Anti-SCML1 primary antibody (H00006322-D01, Abnova, Taipei, Taiwan) working solution was then dropped onto the samples and incubated at 4°C overnight. The cells were washed five time with PBS for 5 min each, and biotinylated goat anti-rabbit IgG secondary antibody (BA1003, Boster, Wuhan, China) working solution was then dropped onto the cells and incubated at 37°C for 30 min.

The cells were washed three times with PBS for 5 min each, followed by streptavidin–biotin complex at 37°C for 30 min. After four washes with PBS for 5 min each, DAB solution (AR1022, Boster) was added at room temperature for staining. The staining time was controlled by microscopic observation and was terminated when the cells developed a brown-yellow color. The cells were washed twice with distilled water and the nucleus was counterstained with hematoxylin. The hematoxylin was then removed and the cells were washed with distilled water until the hematoxylin solution had been totally removed. The coverslips were then dried and mounted with neutral gel, and the cells were observed and photographed under a light microscope (Eclipse 80i, Nikon, Tokyo, Japan).

Electrophoretic Mobility Shift Assay (EMSA): An SCML1 prokaryotic expression vector was constructed for RNA-EMSA. Proteins were expressed and nuclear proteins were extracted and purified. The wild-type probe was biotin-labeled but the other probes were not labeled. The reaction systems were prepared according to the specific experiments. After completion of the binding reaction, the mixture was left to stand at room temperature for 20 min and a 6.5% non-denaturing polyacrylamide gel was prepared. The samples were mixed with EMSA/gel-shift loading buffer and subjected to electrophoresis at 10 V/cm. Electrophoresis was terminated when the bromophenol blue band reached the lower quarter of the gel. The gel was transferred to a PDVF membrane using a wet electrotransfer device with 0.5× Tris-Borate-EDTA for 60 min at 4°C and 380 mA (about 100 V). UV cross-linking was then performed for 45–60 s at a wavelength of 254 nm and 120 mJ/cm^2. After thorough blocking and washing, a chemiluminescent reaction was performed using BeyoECL Plus reagent for 3 min at room temperature, followed by routine X-radiography.

Co-immunoprecipitation: Cell lysates and supernatants were collected and a portion of the cell lysate was used as the input sample. The remaining supernatants were placed in clean centrifugal tubes as the IP sample. A/G beads (200 μl) were washed with pre-cooled PBS and then divided into an IP-target antibody group (160 μl plus 2 μg target antibody and 2 ml PBS) and an IP-lgG group (80 μl plus 1 μg IgG antibody and 1 ml PBS), and incubated for 2–4 h at 4°C. The cells were then centrifuged at 4000 × g at 4°C for 1 min, and the supernatants were discarded. The beads were

washed three times. The pre-prepared cell extracts were then added to the IP-target antibody and IP-IgG groups and incubated overnight at 4°C, followed by the addition of protease inhibitors and phenylmethylsulfonyl fluoride, and centrifugation at 4000 × g at 4°C for 1 min. The beads were then washed three times with pre-cooled PBS. The IP-target antibody group was divided into an IP group and MS group at a ratio of 1:4. Loading buffer was added to the Input, IP, and IgG groups and boiled for 10 min. CHAPS lysate was then added to the MS group and shaken for 30 min, and the supernatants were collected. The lysis procedure was repeated and the cells were then collected. The samples were stored at −20°C for MS detection and silver staining.

Chromatin IP (ChIP): The cells were first cross-linked and sonicated. For IP, lysed positive control, negative control, and experimental samples were prepared and 900 µl of dilution buffer containing protease inhibitor was added to each tube (100 µl), followed by 60 µl protein A/G agarose (50% suspension) and gentle shaking in a water bath at 4°C for 1 h. The agarose beads were then separated using centrifugation at 3000–5000 × g at 4°C for 1 min, and 100 µl of the supernatants were collected and stored at 4°C as the input sample. The remaining supernatants were placed in clean EP tubes and 1 µg anti-RNA polymerase, 1 µg normal mouse IgG, and 1 µg specific antibody were added to the positive control, negative control, and experimental groups, respectively. After gentle shaking at 4°C overnight, 60 µl of protein A/G agarose was added and incubated at 4°C for 1 h. The agarose beads were then precipitated by centrifugation at 3000–5000 × g at 4°C for 1 min and the supernatants were removed. The DNA-protein complexes were then eluted, de-cross-linked, and the DNAs were purified.

Chromatin Isolation by RNA Purification (ChIRP): ChIRP was performed using a ChIRP kit (BersinBio). ChIRP probes and probe sets targeting the investigated genes were designed, synthesized, and biotin-labeled. Cells were harvested, cross-linked, pelleted, lysed, and sonicated, and 1 ml of cell lysate was then subjected to ChIRP. Each sample was transferred into two tubes (0.5 ml per tube) labeled ‘ChIRP odd’ and ‘ChIRP even’, respectively, and 1 ml of hybridization buffer was added to each tube. The prepared odd and even probes were added to the corresponding tubes and hybridized with the cell lysates in a vertical mixer, as follows: denaturing at 65°C for

10 min, hybridization at 25°C for 30 min, denaturing at 50°C for 5 min, and hybridization at 25°C for 90 min. The prepared magnetic beads were added to the hybridization system (2/5 the volume added to normal control group compared with the ChIRP group) and mixed, and binding was carried out at 25°C for 60 min. The magnetic beads were collected and supernatants were discarded, and 1 ml of pre-warmed wash buffer was then mixed with the magnetic beads at 25°C for 5 min. The magnetic beads were collected and supernatants were discarded. The procedure was repeated four times and the DNAs were isolated.

Subcellular Localization of HN Peptides Detected by MitoTracker Staining in Combination with an Immunofluorescence Assay: Cells were seeded on coverslips in 12-well plates and cultured for 24 h to 70% confluence. The medium was discarded and 37°C pre-warmed medium containing MitoTracker® probe (M7512, Invitrogen) was added and staining was carried out by incubation at 37°C and 5% CO_2. The cells were then washed with fresh 37°C pre-warmed medium, and the medium was replaced with growth medium containing 3.7% formaldehyde at 37°C for 15 min to fix the cells. The fixation solution was discarded and the cells were washed twice with PBS for 5 min each. Nuclei were counterstained with DAPI (AR1176, Boster) and the cells were then washed with PBS. Finally, the coverslips were removed from the 12-well plates, air-dried, sealed with anti-fluorescein attenuating sealant (AR1109, Boster), and photographed with a laser confocal microscope (SP8, Leica, Solms, Germany).

Detection of Reactive Oxygen Species (ROS): ROS detection was performed after cell transfection using a ROS detection kit (Beyotime), according to the manufacturer's instructions. The concentration of the detected cells was adjusted to 5×10^5/ml. The working solution was prepared by mixing 2',7'-dichlorodihydrofluorescein diacetate (DCFH-DA) and serum-free DMEM at a ratio of 1:1000 to give a final concentration of 10 μmol/l. The original medium was removed and the cells were washed twice with serum-free medium, followed by adding 1 ml DCFH-DA working solution and mixing. The cells were incubated for 20 min at 37°C and then pelleted by centrifugation at 94 g at 4°C for 3–4 min. The supernatants were discarded and the cells were washed two or three times with serum-free DMEM and then resuspended

in 0.5 ml of serum-free DMEM medium and analyzed using FCM (Millipore).

Detection of Mitochondrial Membrane Potential (MMP) using the JC-1 Assay: MMP was detected using a JC-1 MMP Assay Kit (Beyotime) after cell transfection, according to the manufacturer's instructions. The tested cells were adjusted to 5×10^5/ml. The working solution was prepared by diluting an appropriate amount of JC-1 (200×) with 8 ml ultrapure water per 50 μl of JC-1 (200×). JC-1 was fully dissolved and mixed by vortexing, and 2 ml JC-1 staining buffer (5×) was then added and mixed to give the JC-1 working solution. One milliliter of the working solution was then added to the cell suspension and inverted several times to mix, and then incubated at 37°C for 20 min. An appropriate amount of JC-1 staining buffer (1×) was prepared by adding 4 ml of distilled water per 1 ml of JC-1 staining buffer (5×) and placed in an ice bath. After incubation of the cells at 37°C, they were pelleted by centrifugation at 600 × g at 4°C for 3–4 min. The supernatants were discarded, and the cell pellet was washed twice with JC-1 staining buffer (1×) and resuspended in 1 ml of JC-1 staining buffer (1×). The procedure was repeated twice and the cells were resuspended in JC-1 staining buffer (1×) and analyzed with FCM (Millipore).

Detection of Adenosine 5'-triphosphate (ATP): ATP levels were measured using an enhanced ATP assay kit (Beyotime). After transfection for 48 h, 200 μl ATP detection lysis buffer was added to each well of a six-well plate and the cells were lysed fully by repeatedly pipetting or shaking the plate, followed by centrifugation at 12,000 × g at 4°C for 5 min. The supernatant was collected for subsequent use. The ATP-detection working solution was prepared by diluting the ATP-detection reagent with specific dilution buffer at a ratio of 1:5 (v/v). One hundred microliters of the working solution was then added to the detection wells for 3–5 min at room temperature, followed by 20 μl of the samples, and mixed quickly. Relative light unit (RLU) values were measured using a luminometer (BioTex).

Antibodies: The following antibodies were used: MLH3, P-p53, CHEK2, P-CHEK2, CDC25A, Rb, P-Rb, and P-E2F1 (Affinity, Cincinnati, OH, USA); SCML1 (Abnova, Taipei, China); ESRP1 (Proteintech, Chicago, IL, USA); Bax, Bcl-2, caspase-9, E-cadherin, PCNA, PTEN, CCND1, CDK2, E2F1, MSH2, p53, POLE, P-CDC25A,

LIG4, m6a, and ubiquitin (Abcam, Cambridge, UK).

Primers, Probes, and siRNAs Used in This Study:

Gene	Primer(sense)	Primer(anti-sense)
circNOL10	CAACTCAGGCATGCTTCTGA	TATGGTTCCTGTGGCAAACA
Pre-NOL10	TCCTGCCCTTGGAAAACAGA	CAGAGTTGCTTGCACACAGT
NOL10 mRNA	GGAGGTCCCGGGATTACG	TGTAGCGCTCTCTTCTTCCT
GAPDH	AACGGATTTGGTCGTATTGGG	CCTGGAAGATGGTGATGGGAT
HN1	TGGACACAGACTTGCCAGG	GACAGCGTTCAGGACAGTCA
HN2	GCAAACCTTACCCCACCTGT	CTGAACTTAAAAGTCTCTTCACGG
HN3	CAAGTCCCCCGAAACCAGAC	AGGGTCTAAAGAGCTGTCTGTC
HN5	GGGTTGCGGTATCCTGAACT	GGCCTGTTGGCTTGTTTGAA
HN6	AACCTCCGAGCAACCTATGC	ACCCTTGATAGCAGCTACACC
HN8	GCGGTACCCTAACTGTGCAA	AGGGCCTGTGGACTTGTTAAG
HN9	CGTGAAGAGGCGGACATAAT	ATATGTTGCTCGGAGGTTGG
HN10	GACATACGTTCAATGGCCGC	GCTCCATACATAGGGTCTTCTCG
ESRP1	ACGGAGGACTGCAAAGAAGA	CTGACATGAAGCTGCCCATC
MeRIP-primer1	GCAGACAACCCATTTGGTCT	CTTTTTCCCTCCCTCTTTGG
MeRIP-primer2	CACCGTGGCTCATGTCTGTA	ATCTTGGCTCACTGCAACCT
MeRIP-primer3	GATTTTGATGGGAAGCCAGA	TGTTCCGACTGTGCTTTCAG

siRNA	Primer(forward)	Primer(reverse)
scramble	UUCUCCGAACGUGUCACGU dTdT	ACGUGACACGUUCGGAGAA dTdT
circNOL10 siRNA1	CAUUCCAUUCUGAAGUUUA UU	UAAACUUCAGAAUGGAAUG UU
circNOL10 siRNA2	UUACAUUCCAUUCUGAAGU UU	ACUUCAGAAUGGAAUGUAA UU
circNOL10 siRNA3	CUAUUACAUUCCAUUCUGA UU	UCAGAAUGGAAUGUAAUAG UU
SCML1 siRNA1	ACAAACAGAUCCUCUUGCA UU	UGCAAGAGGAUCUGUUUGU UU
SCML1 siRNA2	GCACCACCUUCAGUUACAA UU	UUGUAACUGAAGGUGGUGC UU
SCML1 siRNA3	UGACCGACUUAAACAAGGA UU	UCCUUGUUUAAGUCGGUCA UU
ESRP1 siRNA1	GCUAGGCUCGGAUGAGAAG UU	CUUCUCAUCCGAGCCUAGC UU
ESRP1 siRNA2	GCAGCUACAUGUAGUUAGU UU	ACUAACUACAUGUAGCUGC UU
ESRP1 siRNA3	GAUAGGCAGUAAUGCUUAC UU	GUAAGCAUUACUGCCUAUC UU

Probe name	Probe sequence
RAP Group-1（Pre-NOL10）	AATTTAGTGACATTATTTTGCCGGATTAGAAAGAGTGATTCTCTTCACCTCAACACAGCTATGGTACTCA
RAP Group-2（Pre-NOL10）	TCTATCAGATAATGGTGTACTCCCTAAAACAGCAATCTTTTCTTTTCTAGACGAGATCAAAGTATGC
RAP Group-3（Pre-NOL10）	GCAGCAAAGTATCTGCTTGTGCCAATCTACTTGAGCGGAAGAACCAATTTTAAAGGACATGAAAATA
RAP Group-4（Pre-NOL10）	AAGTGCTAAAGTCAAACATAAGTAAGTGGAAAACAATCAAGTACAATACAATGGGATTAGTGCCAAAACAGAA
RAP Group-5（Pre-NOL10）	AATTTAAATCCAGGTCTAACCAGAAATCTGTTACCCTTACTGTGAGGCCATGATTCTTTTTGGGAAAC
RAP Group-6（Pre-NOL10）	CAAAACAGAACTCCACACTTTTTCCCCATAAAATTAGGTCTTTTACCTTCCATAAGATGTCTTATCTTTCCG
RAP Group-7（Pre-NOL10）	TCATGTCTTAACTCATTTTCCTTGCTCACATTCACAAGTTTGTCACAATCTTTTCCGTGTATAGCTTT
RAP Group-8（Pre-NOL10）	TCGGCCTCCCAGAGTGCCTTTCTATATTTTAATAGTAGTAGCAGAAAAGGGCAAATGCAAAATTTCAATC
RAP Group-9（Pre-NOL10）	AGATTATACATGTCTATCTCCAAATTTAGCACCATACTAACAGAAGCCAAAGATATCACTCCTAAATGAGTCA
RAP Group-10（Pre-NOL10）	AATAACAATGACTCTAAGCAATGTTAAAGTCCTCTACTTCTACCCTTTTCCTCTCCCAATTTCCTAACTACAC
circNOL10-FISH	CTATAAACTTCAGAATGGAATGTAATAGA
MTRNR2L2-Probe-1	ATCACCTCTAGCATTATCAGTATTAGAGGCAC
MTRNR2L2-Mutant competitor-1	ATCACCTCTATACCGCGGCTACTTAGAGGCAC
MTRNR2L2-Probe-2	CCGGCGTAAAGAGTGTTTTAGATCAATTCCCC
MTRNR2L2-Mutant competitor-2	CCGGCGTAAAACCGCCGGCCACTCAATTCCCC
MTRNR2L8-Probe-1	ATCACCTCTAGCATTCTCAGTATTAGAGGCAC
MTRNR2L8-Mutant competitor-1	ATCACCTCTATACCGCGGCTACTTAGAGGCAC
MTRNR2L8-Probe-2	CCGGCATAAAGAGTGTTTTAGATCAATTCCCC
MTRNR2L8-Mutant competitor-2	CCGGCATAAAACCGCCGGCCACTCAATTCCCC
